# Bioinspired Molecular Factories with Architecture and In Vivo Functionalities as Cell Mimics

**DOI:** 10.1002/advs.201901923

**Published:** 2020-01-09

**Authors:** Tomaž Einfalt, Martina Garni, Dominik Witzigmann, Sandro Sieber, Niklaus Baltisberger, Jörg Huwyler, Wolfgang Meier, Cornelia G. Palivan

**Affiliations:** ^1^ Department of Chemistry University of Basel Mattenstrasse 24a, BPR 1096, P.O. Box 3350 CH‐4002 Basel Switzerland; ^2^ Department of Pharmaceutical Sciences Division of Pharmaceutical Technology University of Basel Klingelbergstrasse 50 CH‐4056 Basel Switzerland

**Keywords:** amphiphilic polymers, artificial cells, enzymes, molecular factory, polymersomes

## Abstract

Despite huge need in the medical domain and significant development efforts, artificial cells to date have limited composition and functionality. Although some artificial cells have proven successful for producing therapeutics or performing in vitro specific reactions, they have not been investigated in vivo to determine whether they preserve their architecture and functionality while avoiding toxicity. Here, these limitations are overcome and customizable cell mimic is achieved—molecular factories (MFs)—by supplementing giant plasma membrane vesicles derived from donor cells with nanometer‐sized artificial organelles (AOs). MFs inherit the donor cell's natural cytoplasm and membrane, while the AOs house reactive components and provide cell‐like architecture and functionality. It is demonstrated that reactions inside AOs take place in a close‐to‐nature environment due to the unprecedented level of complexity in the composition of the MFs. It is further demonstrated that in a zebrafish vertebrate animal model, these cell mimics show no apparent toxicity and retain their integrity and function. The unique advantages of highly varied composition, multicompartmentalized architecture, and preserved functionality in vivo open new biological avenues ranging from the study of biorelevant processes in robust cell‐like environments to the production of specific bioactive compounds.

## Graphical abstract

Strategy for creating bioinspired molecular factories. Prior formed artificial organelles are integrated together with various cargoes (small molecular weight molecules, biomacromolecules, and combinations of thereof) into giant plasma membrane vesicles generated by donor cells. Transfer of material from the donor cell cytoplasm and membrane is achieved during vesicle formation, resulting in compartment‐in‐compartment architecture of the molecular machinery.

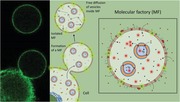

## 1 Introduction

Life is based on an incredibly complex scenario in which biochemical reactions take place in specifically defined microenvironments and compartmentalized spaces. Inspired by nature, various approaches have been used to combine functional molecular units (natural or synthetic) with supramolecular assemblies to develop the first prototypes of artificial cells^1^, ^2^ with the aim of providing micrometer range compartments with cell‐like properties for understanding fundamental biological phenomena and processes, and eventually offering solutions to medical problems.^1^, ^2^, ^3^ Various designs have been used to generate cell‐sized compartments with single specific functions^4^, ^5^, ^6^, ^7^, ^8^, ^9^, ^10^ as a result of inserting functional biomacromolecules (i.e., enzymes, membrane proteins) in a certain type of synthetic template, e.g. lipid‐ or polymer‐based giant unilamellar vesicles,^8^, ^11^, ^12^, ^13^, ^14^ lipid‐coated porous silica nanoparticles,^9^ layer‐by‐layer capsules,^10^ membrane free microdroplets,^15^ or micro‐scale proteinosomes.^5^ However, these simple artificial cells with an intracompartment medium mainly based on buffer‐solutions,^16^ and only recently extended to bacterial cell extracts,^17^, ^18^ lack the multicompartment cell architecture and complexity of cytosolic cell media. A recent advance in the development of artificial cells has been achieved by constructing “compartments‐in‐compartment” microscale structures that are based on encapsulation of nanoscale liposomes/polymersomes inside polymer‐/lipid‐based giant unilamellar vesicles with sizes in the micrometer range.^13^, ^19^ Multicompartmentalization allows biomacromolecules placed in different compartments to take part in two/three step cascade reactions.^13^, ^20^, ^21^ However, the use of simple buffers as internal media inside the compartments, and of organic solvents or emulsions during the preparation procedures, which can affect the biomolecules, represent severe drawbacks of current multicompartment systems.

An elegant strategy to yield compartment systems with more complex composition is based on the generation of cell‐derived giant plasma membrane vesicles (GPMVs).^22^, ^23^, ^24^ The membrane and internal composition directly mirror the composition of the cells from which they originated, except for larger cellular organelles and intracellular structures (e.g., nuclei, Golgi apparatus, vesicles).^25^ Although this is very promising for situations requiring a complex composition of the inner cavity of the compartment, efforts to date have been mainly devoted to their supplementation with small molecular weight compounds, or with biomolecules, such as lipids, or proteins,^26^ and only one study has involved the internal encapsulation of nanoparticles.^27^ There are currently no reports of the creation of functional artificial cells with the outer membrane complexity of a cell, and an internal cytoplasm.

**Figure 1:**
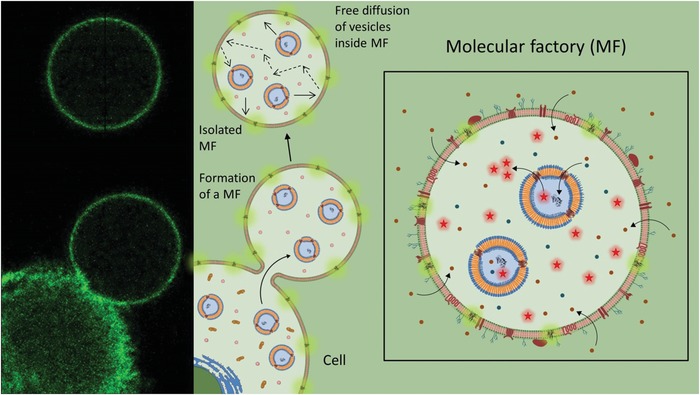
Strategy for creating bioinspired molecular factories. To create a molecular factory (MF) we start by internalizing artificial cargoes (specific molecules and preformed artificial organelles based on polymersomes loaded with active compounds) in the donor mammalian cell. The membrane of the donor cell can also be modified to contain proteins or receptors of interest. Once loaded with all the necessary components of the MF, a transfer of material from the donor cell cytoplasm and membrane is achieved during vesicle formation. After isolation, the MFs are completely independent of the donor cells.

Here, we introduce a general strategy for creating microscale bioinspired molecular factories (MFs) by supplementing GPMVs with the necessary molecules and nanocompartments serving as artificial organelles (AOs) in order to mimic the architecture and functionality of eukaryotic cells, and then evaluate their performance in vivo. AOs are formed by encapsulation of enzymes inside polymersomes equipped with channel porines, prior to their internalization by donor cells. AOs have various advantages, such as an increase in the in situ enzymatic activity by nanoscale confinement, a protection of the enzymes inside their cavity and the possibility to obtain separate reaction spaces by coencapsulation of different AOs when more complex reactions are studied.^28^, ^29^, ^30^ In our approach, donor cells are first preloaded with supplementary cargos (synthetic molecules, nanocompartments and AOs) and simultaneously engineered (genetically if necessary) to overexpress specific biomacromolecules. Then a special “vesicular buffer” is added to the cells and induces formation of GPMVs that are equipped with the functional artificial cargoes, specifically implemented by prior internalization into the donor cells (E‐GPMVs). The hosting by AOs of reactive components, which perform desired reactions, represent the key elements for inducing specific biofunctionality of the MF as they can diffuse and function in situ (**Figure** **1**). Together, the intrinsic composition of GPMVs with the same membrane and cytosol environment as the donor cells and the addition of functional AOs induce an unprecedented complexity of the resulting MFs that supports production of desired compounds/signals in a close‐to‐nature manner. We have assessed these MFs in vivo to study whether they preserve their architecture and functionality, and thus serve as cell‐mimics. As an example of a medical application we engineered MFs to support in situ the enzymatic degradation of H_2_O_2_, which is well known to be involved in oxidative stress, and plays a significant role in many disease states including arthritis, Parkinson's disease, cancer, and AIDS. As these MFs closely represent cell mimics, they are expected to serve as the basis for developing highly efficient therapeutic and diagnostic solutions for which there is a tremendous need to decrease global medical costs.

## 2 Strategy to Create MFs with Architecture and Functionality as Cell Mimics

**Figure 2:**
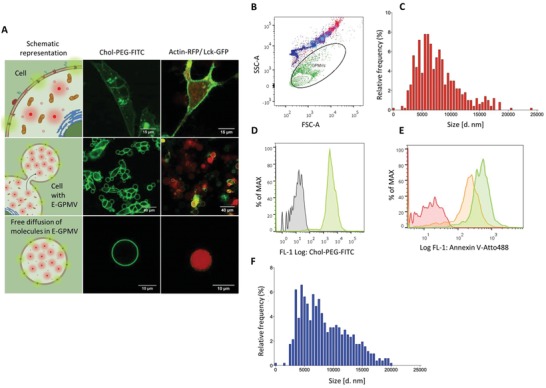
Transfer of cytosolic components from the cellular cytosol into E‐GPMVs. A) Left column: Top) Schematic representation of HepG2 cells with enhanced membrane and cytosolic content. Central) Illustration of the formation of equipped‐GPMV (E‐GPMV) from the donor cells. Bottom) Isolated E‐GPMV. Middle column: Top) CLSM micrograph of HepG2 cells containing Chol‐PEG5000‐FITC copolymer in the membrane (Green), Scale bar = 15 µm. Central) CLSM micrograph of E‐GPMVs formation by several cells, Scale bar = 40 µm; Chol‐PEG5000‐FITC copolymer is inserted both in the cells and in the GPMVs membrane. Bottom) CLSM micrograph with a zoom of one isolated E‐GPMV containing Chol‐PEG5000‐FITC copolymer in the membrane (Scale bar = 10 µm). Right column: Top) CLSM micrograph of HepG2 cells simultaneously containing Actin‐RFP protein (Red) and the membrane protein Lck‐GFP (Green), Scale bar = 15 µm. Central) CLSM micrograph of E‐GPMVs formation by several cells, Scale bar = 40 µm; Actin‐RFP protein (Red) and the membrane protein Lck‐GFP (Green) are located both in the cells and in the GPMVs. Bottom) CLSM micrograph with a zoom of one isolated E‐GPMV containing Actin‐RFP protein (Red) and the membrane protein Lck‐GFP (Green), Scale bar 10 µm. CLSM Controls are available in the Supporting Information. B) Flow Cytometry analysis of E‐GPMVs, size characterization by sideward and forward scattering analysis. The ellipse represents E‐GPMV populations (2.0–9.9 µm) used for analysis by flow cytometry. Blue to red color‐coded polystyrene particles in sizes from 2.0, 3.4, 5.11, 7.6, 9.9, and 14.3 µm. Green: E‐GPMV population. C) Size distribution of the E‐GPMVs with membranes supplemented with the membrane protein Lck‐GFP obtained by analysis of CLSM micrographs. N = 501 individual E‐GPMVs. D) Flow cytometry analysis of E‐GPMVs equipped with Chol‐PEG5000‐FITC copolymer (Green) and control (unmodified) GPMVs (Gray). E) Flow cytometry analysis of Annexin V‐Atto488 binding to E‐GPMVs equipped with the copolymer Chol‐PEG5000: Control GPMVs without Annexin V (Red), control GPMVs with added Annexin V (Green) and E‐GPMVs equipped with Chol‐PEG5000 copolymer and added Annexin V (Orange). F) Size distribution of E‐GPMVs equipped with Chol‐PEG5000‐FITC copolymer obtained by analysis of CLSM micrographs. *N* = 501 individual E‐GPMVs.

Achievement of a close‐to‐nature cell mimic requires the transfer of functional elements (confined AOs, synthetic‐ and biomolecules) necessary to support overall functionality inside GPMVs used as micrometer sized compartments with intrinsic natural composition.^2^ At the same time, the resulting hybrid cell‐derived compartment should simultaneously be stable and allow exchange of molecules with the environment as in a natural cell.^31^ First we produce AOs by a bottom‐up approach in which we equip synthetic nanocompartments (polymersomes) with active compounds (enzymes, proteins, mimics), and simultaneously insert membrane proteins to allow in situ catalytic reactions.^29^ Then we introduce the necessary elements in a human‐derived model cell line, i.e., HepG2 liver carcinoma, by genetic overexpression (e.g., enzymes, proteins) and internalization (e.g., nanocompartments, AOs). After internalization of the nanocompartments and/or artificial organelles, we add a mixture based on a combination of molecules in order to induce the GPMV formation (“vesiculation buffer”). The vesiculation buffer induces a direct budding of the cellular plasma membrane, during which, freely diffusing entities present in the cellular cytosol are transferred to the formed GPMV.^22^ 4–5 h after, the newly equipped‐GPMVs (E‐GPMVs), containing cytosol supplemented with the artificial cargoes, are separated from cell debris by centrifugation and then isolated. The components of the vesiculation buffer are not expected to show toxicity in vivo after E‐GPMV formation, due to their relatively low half‐life.^32^ In order to establish the size distribution of E‐GPMVs, we supplement them with a fluorescent model membrane protein LcK tyrosine kinase‐GFP (Lck‐GFP) by genetically engineering HepG2 cells to overexpress this protein using a baculovirus gene transfer (BacMam 2.0).^33^ When Lck‐GFP is successfully inserted, it preserves its fluorescence property, and does not affect the integrity of the membrane compartment (Figure S1, Supporting Information), in agreement with previous reports about the insertion of gap junction proteins in the membrane of giant plasma membrane vesicles.^26^, ^34^, ^35^ E‐GPMVs are isolated with a size range of 2–20 µm, as demonstrated by confocal laser scanning microscopy (CLSM) and flow cytometry data (**Figure** **2**A–C). Distribution of the resulting E‐GPMV sizes is lognormal with the probability density function: mean 8.84 µm and standard deviation 0.47 µm (*p*‐value = 0.996 according to Kolmogorov–Smirnov test) (Figure S2, Supporting Information).

## 3 E‐GPMVs with Simultaneously Modified Membranes and Cavities

We first tested whether our approach allows supplementing E‐GPMVs with synthetic molecules to improve the mechanical stability of their membranes, a necessary step for overcoming the limited intrinsic stability of giant plasma membrane vesicles. As model compounds for this purpose, we selected a synthetic polymer, cholesterol‐functionalized polyethylene glycol (PEG) 5000 fluorescein isothiocyanate (Chol‐PEG5000‐FITC, 5000 g mol^−1^), which anchors its hydrophobic region in the membranes whilst protecting the membrane surface through steric shielding of the PEG chains (**Figure** **2**A,D).^36^ Successful anchoring of Chol‐PEG5000‐FITC in HepG2 membranes and its transfer to the E‐GPMV membranes was identified by the FITC signal associated with the membrane in CLSM (Figure 2A), and further confirmed by flow cytometry analysis (Figure 2D).

To clarify whether phosphatydilserine was accessible on the outer interface of the membrane, we compared E‐GPMVs with and without modification with Chol‐PEG5000 after staining with Annexin V, a well‐known reporter for phosphatydilserine. Indeed, Chol‐PEG5000 was capable of partially shielding the binding of Annexin V (34 kDa) to the outer interface of the plasma membrane (Figure 2E). In addition, HepG2 derived GPMVs expressed specific HepG2 membrane markers, such as the liver specific asialoglycoprotein receptor (Figure S3, Supporting Information). Insertion of polymer chains in the membrane of E‐GPMVs makes the membrane protein‐repellent (as is the case with PEG), and increases its stability, as in lipid membranes. The sizes determined by CLSM of E‐GPMV‐equipped with Chol‐PEG5000 were fitted with a lognormal function with a probability density function mean of 8.92 µm and standard deviation 0.50 µm (*p*‐value = 0.08 according to Kolmogorov–Smirnov test) (Figure S4, Supporting Information) and complemented by flow cytometry (Figure S5, Supporting Information). E‐GPMVs with Chol‐PEG5000 inserted in their membranes have a larger size range than GPMVs without the polymer (Figure 2F), but the difference of the mean values is not statistically significant (*p*‐value = 0.001, Welch two sample *t*‐test and *p*‐value = 0.005, Wilcoxon rank sum test).

In order to obtain E‐GPMVs with a modified membrane and inner cavity, we simultaneously equipped them with a small hydrophilic fluorophore (CellTracker Deep Red Dye, referred herein as CTDR)^37^ and Chol‐PEG5000‐FITC polymer. First, the fluorescence intensity associated with CTDR alone indicates its successful transfer to the inside of the E‐GPMVs in a concentration dependent manner, in the range 1 × 10^−6^ to 10 × 10^−6^ m (Figure S6, Supporting Information), and is in agreement with the previously reported transfer of low molecular weight compounds present in the cytoplasm of the donor cells.^26^ However, we observed that this process is not linear, but exponential, and thus by controlling the number of desired molecules in the donor cell cytosol, it is possible to control their final amount inside E‐GPMVs, which is a necessary step when reactions have to be implemented inside MFs. Second, when CTDR and the polymer were transferred, it was shown by flow cytometry that more than 90% of the derived compartments presented fluorescence signals associated with both the CTDR fluorophore and the Chol‐PEG5000‐FITC polymer molecules (Figure S7, Supporting Information). In addition, flow cytometry analysis indicated an increase in membrane stability of the compartments simultaneously equipped with Chol‐PEG5000 and CTDR (Figure S5C, Supporting Information) in agreement with the observation for PEGylated lipid vesicles.^38^ Similarly, CDTR and the membrane protein Lck‐GFP were successfully cotransferred to E‐GPMVs and were located inside the cavity and in the membrane, respectively: our approach allows specific localization of the artificial cargos required to support the molecular machinery (Figure S6A, Supporting Information). A necessary step for the feasibility of our strategy to create MFs was to specifically colocalize biomolecules inside GPMVs to further support their internal reactions/functionality. HepG2 cells were treated to overexpress hydrophilic enhanced green fluorescent protein (e‐GFP), a combination of hydrophilic molecules, red fluorescent protein (RFP) and eGFP (RFP/eGFP), and a combination of hydrophilic Actin‐RFP and hydrophobic Lck‐GFP (Actin‐RFP/Lck‐GFP).

The fluorescence signals arising from the compartment cavity or membrane provided an evaluation of the presence of the biomacromolecules (*M*_w_ > 30 kDa) by CLSM micrographs (Figure 2A; Figure S8, Supporting Information). Both the hydrophilic eGFP, and when added in combination with a second hydrophilic protein, RFP, were successfully transferred to cavities of the E‐GPMVs (Figure S8, Supporting Information). Colocalization correlation coefficients for E‐GPMVs loaded with hydrophilic biomolecules eGFP and RFP (PCC = 0.85 ± 0.11, M1 = 0.86 ± 0.21, and M2 = 0.78 ± 0.1) indicate that the proteins were simultaneously transferred and specifically localized in the cavity (Figure S8B, Supporting Information). Similarly, a combination of hydrophilic and hydrophobic proteins (Actin‐FRP and Lck‐GFP, respectively) was transferred to the appropriate locations inside E‐GPMVs, the cavity and the membrane, respectively (Figure 2A).

## 4 E‐GPMVs with Cell‐Specific Subcompartmentalized Architecture

In order to design a multicompartment system equivalent to eukaryotic cells, we envisioned the transfer of nanosized vesicular subcompartments (liposomes and polymersomes) to equipped‐GPMVs, during the passive flow of cytoplasm that gradually fills the blebbing plasma membrane that forms a GPMV. This represents a challenging step because of the need to preserve the integrity of both nano‐compartments and GPMVs. First, we prepared 100–200 nm model liposomes based on DSPC:chol:DSPE‐PEG (48:42:10 mol%) and DSPS:chol:DSPE‐PEG (53:42:5 mol%) by the lipid film rehydration technique and subsequent size adjustment by extrusion. Neutral (DSPC) and negatively charged (DSPS) phospholipids selected to form liposome membranes were enhanced with PEGylated phospholipids (DSPE‐PEG) to increase steric repulsion. This subsequently reduced the expected liposome–GPMV membrane interactions, thereby supporting free movement and preservation of the liposome integrity once in the GPMV cavity. In addition, PEGylated phospholipids (DSPE‐PEG) are expected to increase the fraction of the cell‐integrated liposomes escaping the endosome/lysosome.^39^ A 1 × 10^−3^ m sulforhodamine B (SRB) solution in phosphate‐buffer saline pH 7.4 (PBS) was used during the film rehydration method, resulting in encapsulation of the fluorescent tracker in the liposome cavity. The encapsulated SRB served as a marker for investigating both the integrity of the liposomes and their dynamics inside the GPMVs. After removing nonencapsulated free SRB from prepared SRB‐loaded liposomes by size‐exclusion chromatography, the liposomes were characterized by dynamic light scattering (DLS) and transmission electron microscopy (TEM) (Figure S9, Supporting Information). Vesicular structures were obtained with a hydrodynamic diameter (DH) of 173.9 ± 9.0 nm (DSPS‐based liposomes) and DH of 100.8 ± 1.7 nm (DSPC‐based liposomes) and PDIs of 0.197 ± 0.01 and 0.05 ± 0.01, respectively. Liposomes based on DSPC had a neutral surface charge (ζ = 0.507 ± 0.1 mV), whereas those based on DSPS were slightly negatively charged (ζ = −3.5 ± 0.5 mV). Encapsulation of SRB inside the liposome cavity and successful removal of the nonencapsulated dye were demonstrated by fluorescence correlation spectroscopy (FCS): the free SRB in solution after purification was 4.3% for DSPS‐based liposomes and 0.6% for DSPC‐based liposomes (Figure S9, Supporting Information).

Cellular internalization of liposomes was achieved by incubating HepG2 cells treated to overexpress LcK‐GFP in the presence of both types of liposome at a total lipid concentration of 20 × 10^−3^ m for 24 h. It is noted that PEG does not significantly hinder the unspecific, passive endo‐ or pinocytotic concentration dependent uptake of these stealth liposomes after 24 h.^39^ The HepG2 cells were then extensively washed with culture medium to remove all nonintegrated liposomes. After the vesiculation process of the donor cells, we observed by CLSM micrographs that there was little to no diffuse SRB signal present in the GPMVs (Figure S10, Supporting Information). On contrary, distinct localized fluorescence signal spots corresponding to the SRB‐loaded liposomes were observed, with a completely different aspect than the diffuse fluorescence signal of the low molecular weight fluorophore CDTR (control), which was homogeneously distributed throughout the GPMV cavity (Movies S1 and S2, Supporting Information). The distinct fluorescent spots associated with the liposomes indicate that the transfer process did not affect the integrity of the liposomes inside equipped‐GPMVs.

**Figure 3:**
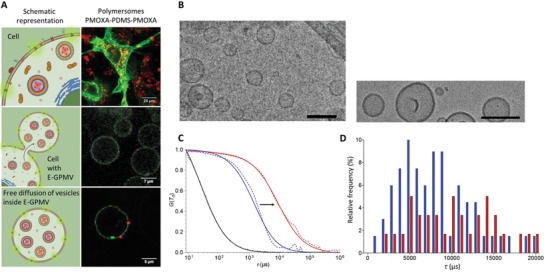
Transfer of cytosolic components from the cellular cytosol into E‐GPMVs. A) Left column: Top) Schematic representation of HepG2 cells with enhanced membrane and cytosolic content. Central) Illustration of the formation of several equipped‐GPMV with multicompartment structure (E‐GPMV) from the donor cells. Bottom) One isolated E‐GPMV with microcompartment structure. Right column: Top) CLSM micrograph of HepG2 cells containing membrane protein LcK‐GFP (Green) and SRB‐loaded PMOXA_6_‐PDMS_44_‐PMOXA_6_ polymersomes (Red), Scale bar 20 µm. Central) CLSM micrograph of several E‐GPMV, Scale bar 7 µm. Bottom) CLSM micrograph of one isolated E‐GPMV simultaneously containing Lck‐GFP protein and SRB‐loaded PMOXA_6_‐PDMS_44_‐PMOXA_6_ polymersomes, Scale bar 5 µm. See Movies S3–S8 in the Supporting Information. CLSM Controls are available in the Supporting Information. B) Cryogenic transmission electron micrograph of PMOXA_6_‐PDMS_44_‐PMOXA_6_ polymersomes in PBS pH 7.4. Scale bar represents 200 nm (left) and 50 nm (right). C) FCS analysis of SRB‐loaded PMOXA_6_‐PDMS_44_‐PMOXA_6_ polymersomes in PBS pH 7.4 (Blue) and SRB‐loaded PMOXA_6_‐PDMS_44_‐PMOXA_6_ polymersomes within E‐GPMVs (Red), Free SRB at pH 7.4 PBS (Black). Arrowhead indicates the change in diffusion time from free SRB polymersomes once they are encapsulated in E‐GPMVs. Dotted line—experimental auto correlation curves, full line—fit. Curves normalized to 1 to facilitate comparison. D) Frequency distribution of diffusion times taken from individual FCS measurements: SRB‐loaded PMOXA_6_‐PDMS_44_‐PMOXA_6_ polymersomes in PBS pH 7.4 (Blue) and SRB‐loaded PMOXA_6_‐PDMS_44_‐PMOXA_6_ polymersomes within E‐GPMVs (Red) (*n* = 67 and *n* = 30 respectively).

In order to improve the stability of the nanocompartments inside equipped‐GPMVs, we used polymeric vesicles (polymersomes) that are known to have higher mechanical stability than liposomes due to their thicker membranes.^40^, ^41^ First, polymersomes based on the amphiphilic poly(2‐methyloxazoline)‐block‐poly(dimethylsiloxane)‐block‐poly(2‐methyloxasoline) (PMOXA_6_‐PDMS_44_‐PMOXA_6_) copolymer were formed using the thin film rehydration method, since this type of polymersome has already been used for the development of catalytic compartments,^42^ and even AOs by encapsulation of enzymes and proteins.^29^, ^43^ We encapsulated fluorescent SRB or carboxyfluorescein (CF) inside polymersome cavities to serve as reporters for the polymersome spherical architecture and membrane integrity. Architecture and size of assemblies were determined by a combination of transmission TEM, Cryo‐TEM, DLS, and FCS (**Figure** **3**; Figure S11, Supporting Information). Polymersomes with *D*_H_ of 187 ± 13 nm, and a net neutral Zeta potential (ζ = −0.15 ± 1.77 mV) were obtained. Successful encapsulation of the fluorescent dye within the polymersomes and purification from nonencapsulated dye, were determined by FCS (free SRB fraction: 10.7%).

**Figure 4:**
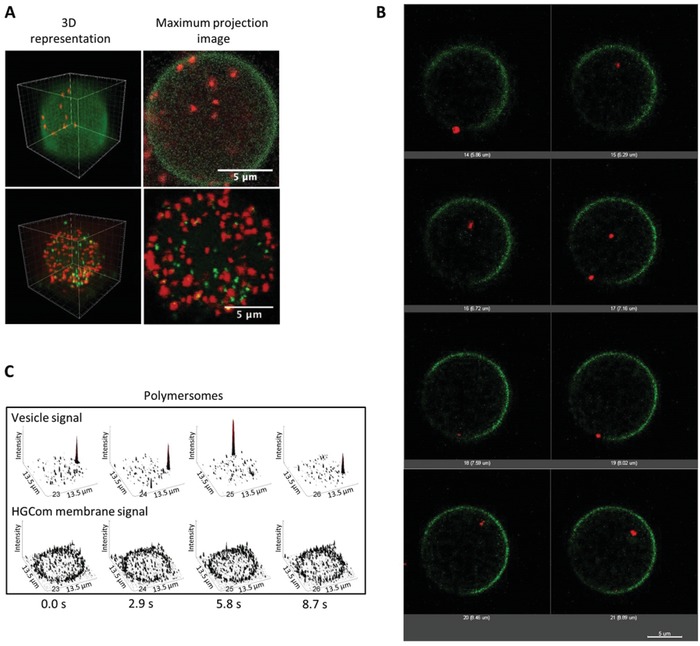
Subcompartmentalization within E‐GPMVs. A) 3D reconstructions (left) and maximum intensity projection images of Z stacks (right) of E‐GPMVs with multicompartment architecture. Top: SRB‐loaded polymersomes inside E‐GPMVs equipped with Lck‐GFP. Red: signal of SRB‐loaded polymersomes. Green: signal of Lck‐GFP membrane protein. Bottom: SRB‐loaded polymersomes and CF‐loaded polymersomes inside E‐GPMVs. Red: signal of SRB encapsulated inside polymersomes. Green: signal of CF encapsulated inside polymersomes. B) Single plane projections of different Z positions during E‐GPMV Z‐stacking process by CLSM. Z interval = 1 µm. Red: SRB loaded polymersomes. Green: Lck‐GFP membrane protein. C) Single plane recordings of PMOXA_6_‐PDMS_44_‐PMOXA_6_ polymersomes inside E‐GPMVs measured by CLSM. See Movies S3–S8 in the Supporting Information.

PMOXA‐PDMS‐PMOXA polymersomes have been previously reported as cyto‐compatible and capable of successful escape from endosomes with preserved integrity.^44^ Therefore, HepG2 cells were treated to overexpress LcK‐GFP and cultured in the presence of 1 mg mL^−1^ PMOXA_6_‐PDMS_44_‐PMOXA_6_ polymersome solution to promote their up‐take.^29^, ^41^ Uptake of SRB‐loaded polymersomes into HepG2 cells prior to the vesiculation process, and their subsequent transfer from the cellular cytoplasm into E‐GPMV cavities was evaluated by CLSM (**Figures** 3A and **4**; Figure S12G–I, Supporting Information). Whilst LcK‐GFP was inserted in the E‐GPMV membrane, the polymersomes were encapsulated inside the E‐GPMVs cavity (Figure 4A–C), and their architecture was preserved even after storage at 20 °C for 7 days (Figure S12, Supporting Information). Distinctly localized fluorescence spots associated with the SRB‐loaded polymersomes within the E‐GPMV cavity, with negligible fluorescence spreading, were observed by CLSM. The long‐term stability of polymersomes inside E‐GPMVs (7 days) supports creation of a robust multicompartment cell‐like architecture. In addition to CLSM, transmission electron microscopy analysis of E‐GPMVs revealed polymersome‐like structures within ruptured E‐GPMVs (Figure S13, Supporting Information). The escape of polymersomes from the endosomes and their preserved architecture inside the isolated E‐GPMVs were facilitated by the change in the intracellular membrane structures (i.e., lysosomal, endosomal, organelles) induced during E‐GPMV formation. Indeed, upon incubating the cells for 1 min with Chol‐PEG‐Cy5, the fluorophore rapidly accumulated in the intracellular membrane structures (Figure S14A, Supporting Information), and dissipation of the fluorescence signal of Chol‐PEG‐Cy5 upon vesiculation (Figure S14B, Supporting Information) suggests that E‐GPMV formation induces changes in intracellular membranes, which thus facilitate escape of polymersomes.

Interestingly, unlike PEG shielded liposomes, which moved freely inside E‐GPMVs cavity, polymersomes moved along the membrane, which might indicate a slight interaction with the inner interface of the GPMV membrane (Figure 4; Figure S12B,C, Figure S15, and Movie S3–S7, Supporting Information). This behavior has been observed immediately after the polymersomes transfer to the cavity of the GPMVs, while these later were still attached to the donor cells (Movies S4 and S7, Supporting Information). However, their rapid movement at the membrane‐cavity interface indicates that this interaction is minor and does not affect their mobility. Besides, polymersomes did not escape from the cavity of E‐GPMVs as indicated by the maximum intensity projection images of Z stacks of CLSM micrographs (Figure 4B). 3D reconstruction of the Z stacks of these CLSM micrographs indicates that there was an efficient transfer of polymersomes into E‐GPMVs. Furthermore, this was concentration dependent as observed by incubating HepG2 cells in the presence of increasing polymersome concentrations in the range 0.5–3 mg mL^−1^ (Figure S16, Movie S7, Supporting Information). The number of polymersomes/E‐GPMV is described by a geometrical discrete distribution (Figure S17A, Supporting Information). The average number of polymersomes/E‐GPMVs obtained in single plane projections of CLSM micrographs as function of the E‐GPMVs size was statistically analyzed by ordinary least square fitting (OLS) (Figure S17B, Supporting Information). The best fit was obtained with a log–log formula (Supporting Information): *ln* (*N*_particle_) =  *a* *ln*(*Size*_particle_) + *b* *Size*_particle_ with parameters a = 1.673 and b = −0.142 (adjusted R‐squared = 0.9872). By increasing the initial concentration of polymersomes to be up‐taken by the donor cells from 0.5 to 3 mg mL^−1^, the percentage of polymersome‐loaded E‐GPMVs increased from 25%, to 89%. Each cell loaded with an initial polymersomes concentration of 0.5 mg mL^−1^ yielded 1.5 ± 5 E‐GPMVs, whereas 3.6 ± 1.3 E‐GPMVs were generated for an initial polymersome concentration of 3 mg mL^−1^. When SRB‐loaded polymersomes and CF‐loaded polymersomes were cotransferred inside E‐GPMVs, their fluorescence signal did not overlap. The increased loading of GPMVs with increasing initial polymersome concentration and cotransfer clearly indicate that the polymersomes preserved their integrity and thus opens the possibility of a multicompartment system with different types of nanocompartments.

**Figure 5:**
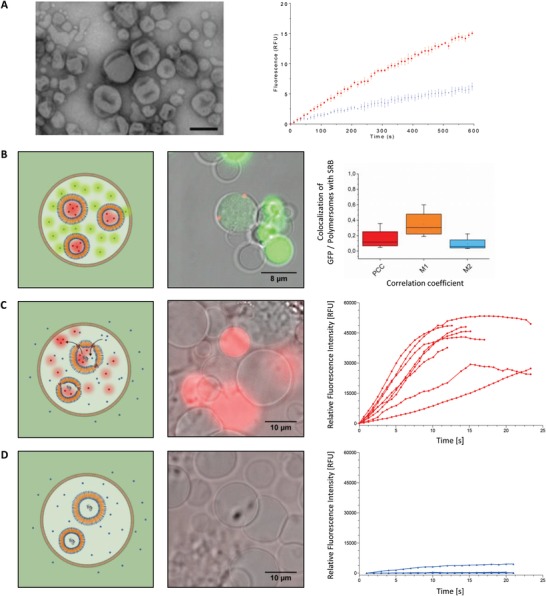
Engineering a MF. A) Left: TEM micrograph of AOs based on PMOXA_6_‐PDMS_44_‐PMOXA_6_ polymersomes loaded with horseradish peroxidase (HRP) and equipped with OmpF. Scale bar: 500 nm. Right: Amplex UltraRed conversion kinetics of AOs equipped with OmpF (red) and AOs without OmpF (blue). Standard deviations are based on 3 individual measurements. B) Spatial colocalization in E‐GPMVs. Left: Schematic representation of E‐GPMVs containing SRB‐loaded polymersomes and cytosolic GFP protein. Middle: CLSM micrograph of E‐GPMVs containing SRB‐loaded polymersomes (Red signal) and cytosolic GFP protein (Green signal). Right: Colocalization analysis of SRB‐loaded polymersomes and GFP protein within E‐GPMVs. PCC = 0.16 ± 0.14, M1 = 0.35 ± 0.1, M2 = 0.09 ± 0.09. *n* = 5 independent CLSM images. C) Functional MFs containing AOs, which convert in situ Amplex UltraRed into resorufin‐like product. Left: Schematic representation of MFs containing AOs. Middle: CLSM micrograph of real‐time production of the resorufin‐like product by AOs inside MFs (Red signal). Right: Relative fluorescence intensity recordings of individual MFs equipped with AOs. See Movie S9, Panel (D) in the Supporting Information) Control MFs equipped with AOs with no OmpF. Left: Schematic representation of E‐GPMVs containing AOs without OmpF. Middle: CLSM Micrograph demonstrating the absence of conversion of Amplex UltraRed into the resorufin‐like product when AOs inside MFs have no OmpF. Right: Relative fluorescence intensity recordings of individual MFs loaded with AOs without OmpF; See Movie S10 in the Supporting Information.

The integrity of polymersomes inside E‐GPMVs, and the exclusion of autofluorescence was also assessed by FCS. Diffusion times of free SRB‐loaded polymersomes in GPMV formation buffer (τ_d_ Q1 = 5209.2 µs, Median = 8445.7 µs) were compared to compartmentalized SRB‐loaded polymersomes in GPMVs (τ_d_: Q1 = 9648.5 µs, Median = 20994.8 µs) (Figure 3C,D). Median values are based on 60 individual measurements of SRB‐loaded polymersomes within an E‐GPMV and SRB loaded polymersomes free in solution (PBS, pH 7.4), respectively. The shift to longer diffusion times for polymersomes inside E‐GPMVs cavity is expected, because of the higher viscosity of the cytosol compared to buffer conditions. The free SRB fraction in solution (after polymersome preparation and purification) and that within E‐GPMVs were determined by FCS. We used a two‐component fit, where the diffusion time of the free SRB in solution (τ_d_: *Q*_1_ = 24.4 µs Median = 27.2 µs) was fixed as the first component. Free SRB fractions were small both after polymersome purification and inside E‐GPMVs (Fraction_SRB_ = 9.04% for polymersomes in E‐GPMVs and 10.7% for free polymersomes) (Figures 3C,D, and 4). To further demonstrate the high degree of control of the inner composition of E‐GPMVs, we transferred eGFP together with SRB‐loaded PMOXA_6_‐PDMS_44_‐PMOXA_6_ polymersomes into E‐GPMVs. The separate localization of the fluorescence signals of SRB and eGFP indicates that individual subcompartments can exist within E‐GPMVs with distinct nano‐environments, which are different from the rest of the E‐GPMVs (**Figure** **5**A; Movie S8, Supporting Information). This strengthens our findings of a multicompartment architecture within E‐GPMVs based on the values of the colocalization coefficients (Figure 5A). For further applications, the variation of the number of polymersomes/E‐GPMVs can be reduced by fluorescence activated cell sorting (FACS) of E‐GPMVs.^45^

For the first time giant plasma membrane vesicles have been equipped with synthetic nanocompartments together with the desired biomolecules to generate subcompartimentalized architecture, which is characteristic of cells. The size of the encapsulated subcompartments can be modified prior to their up‐take by the donor cells depending on the applications.

## 5 MF with Architecture and Functionality as Cell Mimics

To produce in situ AOs, we first preloaded polymersomes with specific biomolecules (enzymes, membrane proteins). We used AOs based on PMOXA_6_‐PDMS_44_‐PMOXA_6_ polymersomes combined with membrane proteins and enzymes, since they have already been reported to preserve their functionality in cells and in vivo.^29^, ^43^ Spatial localization of enzymes within AOs increases the enzyme‐substrate affinity.^28^ As a model we loaded horseradish peroxidase (HRP) into the cavities of PMOXA_6_‐PDMS_44_‐PMOXA_6_ polymersomes that contained the bacterial outer membrane protein F (OmpF) in the membrane to control passage of specific substrates and products. We selected HRP because of its biological role in detoxification of H_2_O_2_, known as toxic by‐products of mitochondrial respiration in eukaryotic cells, and its role in modulating the protein function of by thiol oxidation.^46^, ^47^ More recently, HRP has also gained significant interest in cancer research. Thus, its stability and bioversatility have made HRP one of the most studied enzymes. Prior to incorporation, the purities of HRP and OmpF were determined by SDS‐gel electrophoresis (Figure S18A, Supporting Information). OmpF was selected to render permeable the membrane of the polymersomes because, as a channel porin, it allows the diffusion of small hydrophilic molecules.^48^ Previously inserted into the membrane of PMOXA‐PDMS‐PMOXA polymersomes, OmpF served as “gate” for an orientation independent molecular flow.^42^, ^49^ HRP is able to convert H_2_O_2_, a well‐known harmful compound involved in oxidative stress, and it will thus indicate whether our MFs could have efficient medical applications. Both excess OmpF and nonencapsulated HRP are removed from the prepared AOs by dialysis. These AOs were characterized by DLS and TEM in terms of architecture and size (**Figure** **5**A; Figure S18B–D, Supporting Information). As expected, TEM micrographs show a typical deflated structure for the polymersomes because the measurements are carried out under vacuum. Enzyme encapsulation and membrane protein insertion did not affect the self‐assembly process, and resulted in polymersomes with a *D*_H_ of 122 ± 1.6 nm. We used the formation of the fluorescent enzymatic resorufin‐like product to characterize AOs in terms of functionality prior to their uptake by the donor cells (Figure 5A), and compared them with OmpF‐deficient HRP‐loaded polymersomes. It should be noted that OmpF permits diffusion of molecules with a molecular cutoff of about 600 Da.^50^

In order to create a compartments‐in‐compartment architecture inside MFs, HepG2 cells were incubated with AOs (1 mg mL^−1^ polymer concentration) for 24 h to allow sufficient cellular uptake (Figure 5B,C). The distribution of the polymersomes transfer to E‐GPMV was observed to be concentration dependent. At an initial polymer concentration of 1 mg mL^−1^, E‐GPMVs contained an average of 1.8 ± 1 polymersomes/GPMV (Figure S16, Supporting Information). AOs based on encapsulation of HRP in similar conditions as we used here were reported to contain 6 ± 2 enzymes.^30^ Therefore we calculated that MFs encapsulated a range of 4–24 enzymes/MF. The relative fluorescence intensity of the resorufin‐like product inside individual E‐GPMVs when the encapsulated AOs were equipped with OmpF (Figure 5C), and when deficient of OmpF (Figure 5D), allowed the functionality of the MFs to be established. Amplex UltraRed was chosen as an enzymatic substrate to evaluate whether AOs preserved their functionality inside MF cavities because it can readily diffuse through their lipid membrane, but not through the polymersome membranes, unless they are equipped with OmpF pores.^29^, ^51^ Permeabilization of AOs by OmpF allowed the nonfluorescent substrate Amplex UltraRed to reach the encapsulated enzyme HRP within the cavity where it was converted to a fluorescent resorufin‐like product in the presence of H_2_O_2_.^51^ As the in situ enzymatic activity inside polymersomes was preserved in physiological media, such as human serum,^30^ the activity of AOs is not affected by their encapsulation inside E‐GPMVs. Therefore, the reaction rate limiting step is no longer determined by enzymatic affinity, but by the diffusion of H_2_O_2_ through the lipid membrane of the MFs.^52^, ^53^, ^54^ As the fluorescent resorufin‐like product rapidly diffuses through the MF membrane into its environment, it is difficult to follow the reaction intercompartmentally. To circumvent this limitation for characterization purposes, we used photo‐illumination to locally increase the amount of reactive oxygen species inside the MFs by irradiation with a λ = 543 nm laser after Amplex UltraRed accumulated inside the MFs.^55^ Due to the reaction with H_2_O_2_, the conversion of Amplex UltraRed to the resorufin‐like product was visualized in presence of HRP, and a significant increase in the intensity of the fluorescence signal associated with the reaction product was observed for AOs inside MFs. Note that this increase in the concentration of the fluorescent product was expected due to the confinement of multiple AOs within GPMVs. As expected, the fluorescence within MFs subsequently decreased after ceasing irradiation (Movie S9, Supporting Information). As expected, there is a difference in the fluorescence intensity inside the core of MFs due to the AOs degree of transfer, which is inducing as slight variation of the enzymatic substrate turnover. MFs loaded with polymersomes containing HRP but without OmpF and MFs loaded only with cytoplasm showed only a minor or no increase in fluorescence, because the reaction substrate did not diffuse through the polymersome membranes without OmpF, and the reaction did not take place without the enzyme (auto‐oxidation of substrate is only minor), respectively^51^, ^55^ (Figure 5; Figure S19, Movies S9 and S10, Supporting Information). In addition, in the absence of AOs inside MFs, Amplex UltraRed was not converted into the fluorescent resorufin‐like product (Figure 5; Figure S19, Supporting Information). Thus, we have demonstrated that a substrate able to diffuse through the membrane of E‐GPMVs and inner AOs are necessary elements for MFs to be functional in a cell‐like manner. As PMOXA‐PDMS‐PMOXA‐based AOs have been previously reported to retain their activity for 7 days of storage and even preserve their integrity upon escape from endosomal and lysosomal compartments,^29^, ^43^, ^56^ the only limiting factor for the activity of MFs is the stability of the outer E‐GPMV membrane. Reactions inside AOs freely moving in a complex medium, as in the cytoplasm of E‐GPMVs, have neither previously been achieved in the field of giant plasma membrane vesicles, nor in that of artificial cells developed by bottom‐up approaches.

## 6 In Vivo Evaluation of MF Functionality

**Figure 6:**
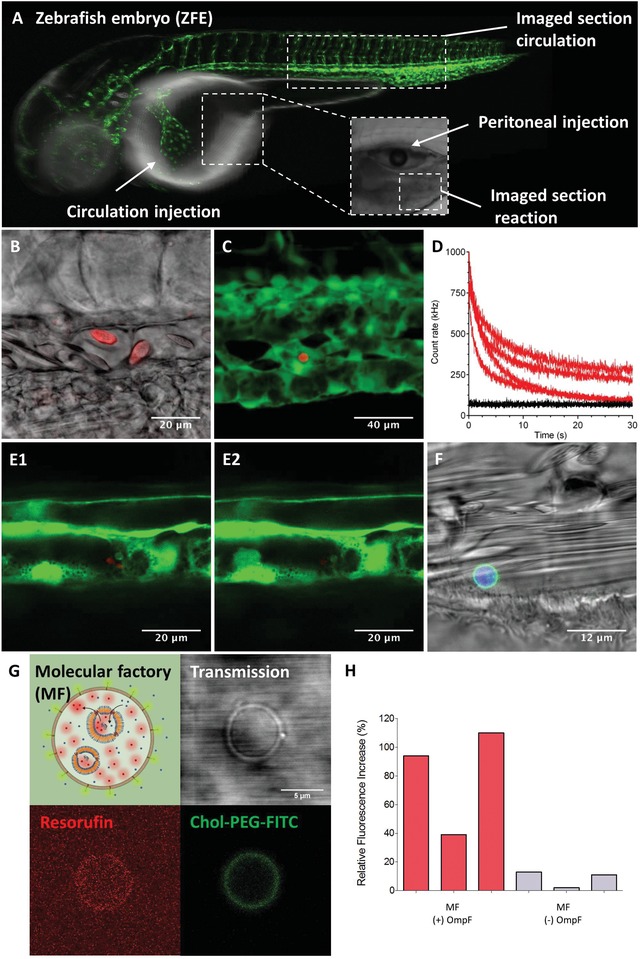
MF functionality in vivo. A) Schematic representation of ZFE injection and imaging. Green: ZFE vasculature. B) RFP‐loaded, Chol‐PEG500 enhanced MFs in ZFE circulation. Red: RFP in MF cavity. C) RFP‐loaded Chol‐PEG5000‐MFs (Red) deposited on ZFE vasculature (Green). D) Decay in fluorescence intensity of individual MFs, in ZFE during photo bleaching. Red: RFP signal. Black: Melanocyte signal. E1) CLSM micrograph before photo bleaching. E2) CLSM micrograph after photo bleaching. F) CLSM micrographs of CTDR and Chol‐PEG5000‐FITC MFs deposited in ZFE vasculature. Blue: CTRD signal, Green: Chol‐PEG5000‐FITC signal. See Movies S11–S14 in the Supporting Information. G) In vivo functionality of MFs in ZFE. Red: Resorufin‐like product signal. Green: Chol‐PEG5000‐FITC signal. H) Production of resorufin‐like product by MFs in ZFE. Relative fluorescence intensity increase within an individual MF (single confocal plane) as compared to background fluorescence. Control: Individual MF containing AOs with no OmpF.

Artificial cells that have previously been reported only showed functionality in vitro where they interacted with their environment, among themselves,^5^ or with other eukaryotic/prokaryotic cells^26^ in culture or solid tumor models.^17^ In order to study whether our MFs preserve their integrity and functionality and are nontoxic when injected in vivo, we selected zebrafish embryos (ZFEs) as a vertebrate animal model, which has previously been used to bridge the gap between in vitro studies and studies in mammalian organisms (e.g., rodents).^57^ Due to their high optical transparency and relatively straightforward genetic modification (e.g., fluorescent tagging of immune cells or vasculature), ZFEs serve as appropriate models to determine the in vivo behavior of drugs and nanoparticles,^58^, ^59^ to serve as disease models,^60^ and have recently also been used to visualize the in vivo functionality of AOs.^29^ In order to promote the stability of MFs in vivo and to decrease nonspecific protein adsorption, we supplemented the membrane with either a nonfluorescent or fluorescent derivative of cholesterol‐polyethylene glycol‐5000 as described above (Figure 2). In addition, we loaded their inner cavity with a fluorescent dye, CTDR, or water‐soluble proteins RFP, as markers for the hollow sphere nature of the E‐GPMVs. The exact number of E‐GPMVs injected could neither be fixed nor determined, due to the sedimentation process when E‐GPMVs were aspirated into the microinjection needle and subsequent needle fixation in the micro injector (10–15 min). As both GPMVs and PMOXA‐PDMS‐PMOXA polymersomes have been reported as noncytotoxic,^26^, ^29^ we did not expect toxic effects for their combination as E‐GPMVs or MFs. Indeed, we did not observe acute toxicity, change of behavior, or heart failure in the injected ZFE. Further, for up to 24 h post injection of E‐GPMVs, no malformations, denaturation of tissue fluids or the yolk mass were observed in the ZFE (**Figure** **6**A). Follow up studies estimating the long‐term survival rate of injected ZFE will serve to complete possible toxic effects of E‐GMPVs, but they are beyond the scope of this article. Once injected, the process of the distribution of E‐GPMVs in the vasculature of the organism started. E‐GPMVs with sizes ranging from 2 to 10 µm were observed to be deposited in the endothelium, or circulating in the vasculature of ZFE for up to 8 h (Figure 6B–F; Movies S11 and S12, Supporting Information). Astonishingly, the membrane of individual MFs was sufficiently robust to survive the aspiration/injection procedure, and the presence of shear stress during blood circulation. The hollow sphere architecture of the MFs, and the preservation of its AOs were evaluated by CLMS analysis, and photo bleaching of RFP within the MF cavities, where there was a typical exponential photo bleaching behavior, as compared to ZFE melanocytes^61^, ^62^ (Figure 6B,C; Figure S20, Supporting Information).

To test the functionality of our cell‐like MFs in a living vertebrate model, MFs were injected into the peritoneal cavity (PC) of transgenic zebrafish with green fluorescent vasculature (kdrl:EGFP zebrafish) 4 days post fertilization. The nonvascular injection site was chosen to achieve a better control over the overall reaction process, and to avoid background fluorescence from the reaction of H_2_O_2_ and Amplex UltraRed substrate with ZFE erythrocytes in blood circulation.^29^ Prior to the injection of MFs, we injected CTDR loaded PEG equipped GPMVs into the peritoneal space of ZFE in order to evaluate their stability (Figure S21, Supporting Information). At 6 h post injection individual E‐GPMVs with CTDR were observed inside their cavities. Based on the stability and overall distribution of E‐GPMVs in the peritoneal space of ZFE (Figure S21, Supporting Information), the functionality of MFs injected into the peritoneal space was investigated in the same area at a higher zoom. MFs were preincubated with H_2_O_2_ and the reaction substrate for 2 h in order to provide the required reaction substrates. After their intraperitoneal injection into ZFE, a second addition of the reaction substrate and H_2_O_2_ was performed directly prior to injection into ZFE, to increase the in vivo substrate levels: a direct visualization of the reaction inside injected MFs is thus possible. The ZFE peritoneal space was observed by CLSM micrographs, which indicated an increase in the signal associated with the resorufin‐like product inside injected MFs, whereas the signal of substrates injected in ZFEs without MFs remained at the background level (Figures S22,23, Supporting Information). A statistically significant 81 ± 37% increase in fluorescence compared to the background was observed within MFs if AOs were equipped with OmpF as compared to an 8 ± 5% increase when OmpF was absent (Figure 6G,H; Figure S18, Supporting Information). Statistical significance was determined by the Student's *t*‐test. While we tested the in vivo functionality directly after injection within a 2 h timespan, due to limitations of the animal permission the experiment had to be stopped. We expect that MFs are able to function in vivo for longer periods of time because the substrate of the enzymatic reaction inside AOs is present in the bioenvironment of MFs, and they were still intact 8 h post injection (Figure 6B–F). Overall, these unprecedented findings show the usefulness of our MFs with complex composition as artificial cell mimics in vivo.

## 7 Conclusions

We have introduced customizable MFs as cell mimics that have a natural membrane and cytosol interior combined with AOs that produce desired molecules or signals. Preformed polymersomes taken up by the donor cells are transferred into hybrid giant plasma membrane vesicles, E‐GPMVs, where they preserve their integrity and move along the membrane to give a multicompartment cell‐like architecture; membrane stability of the E‐GPMVs is improved by insertion of polymers. In addition, polymersomes loaded with catalytic compounds, such as enzymes, play the role of AOs and support in situ reactions inside MFs. Compared to previously reported artificial cells based on bottom‐up approaches, our MFs represent an advance in terms of complexity (given by the intrinsic composition of GPMVs supplemented by nano‐objects transfer) and functionality (supported by in situ enzymatic reactions inside AOs) that has been proven in vivo. Such MFs open new avenues for understanding bioreactions and functions in a close‐to‐nature environment. In addition, by systematic optimization of the conditions in which MFs are produced, it is possible to control the amount of synthetic cargoes with which these artificial cells are equipped. This control can be used to favor a specific interaction, reinforce stability, or tune the overall efficiency of the reactions inside the AOs, as an essential step towards medical applications. Further, our experiments with ZFEs show that these MFs are nontoxic, and can successfully persist in vivo while retaining their integrity and functionality. Such MFs are developed in a straightforward manner that can be easily scaled‐up, and thus have potential for development of highly efficient in vitro diagnostic and in vivo therapeutic solutions depending on the specificity of the enzymes loaded inside the AOs.

## Conflict of Interest

The authors declare no conflict of interest.

## Author Contributions

T.E. and M.G. contributed equally to this work. T.E., M.G., C.P., W.M. contributed to the artificial cell concept, experiment planning, and writing the manuscript. T.E. and M.G. contributed equally to GPMV production, isolation and characterization, polymersome preparation and characterization, OmpF production, isolation and characterization, video analysis, and in vivo experiments. J.H. contributed to the in vitro and in vivo experiments and writing of the manuscript. D.W. contributed to the cell culture and FACS experiments, genetic engineering, and liposome preparation. S.S contributed to zebrafish experiments. N.B. contributed to E‐GPMV production, isolation, and characterization.

## Supporting information

Supplementary Information

Supplemental Movie 1

Supplemental Movie 2

Supplemental Movie 3

Supplemental Movie 4

Supplemental Movie 5

Supplemental Movie 6

Supplemental Movie 7

Supplemental Movie 8

Supplemental Movie 9

Supplemental Movie 10

Supplemental Movie 11

Supplemental Movie 12

Supplemental Movie 13

Supplemental Movie 14
