# Supplementary material for: Bioinspired Molecular Factories with Architecture and In Vivo Functionalities as Cell Mimics
Source: Adv Sci (Weinh). 2020 Jan 9;7(4):1901923. doi: 10.1002/advs.201901923 (PMC7029636; doi:10.1002/advs.201901923)
Supplement: Supplementary file 1 — Supplementary Information [file ADVS-7-1901923-s001.pdf]

Copyright WILEY-VCH Verlag GmbH & Co. KGaA, 69469 Weinheim, Germany, 2020.

ADVANCED SCIENCE
Open Access

Supporting Information

for *Adv. Sci.,* DOI: 10.1002/advs.201901923

Bioinspired Molecular Factories with Architecture and In Vivo Functionalities as Cell Mimics

Tomaž Einfalt, Martina Garni, Dominik Witzigmann, Sandro Sieber, Niklaus Baltisberger, Jörg Huwyler, Wolfgang Meier, and Cornelia G. Palivan**

Supporting Information

Bioinspired molecular factories with architecture and *in vivo* functionalities as cell mimics

Tomaž Einfalt[1a,2], *Martina Garni*[1a], *Dominik Witzigmann*[2], *Sandro Sieber*[2], *Niklaus Baltisberger*[1], *Jörg Huwyler*[2], *Wolfgang Meier*[1*], *Cornelia G. Palivan*[1*]

[1]Department of Chemistry, University of Basel, Mattenstrasse 24a, BPR 1096, P.O. Box 3350, CH-4002 Basel, Switzerland
[2]Department of Pharmaceutical Sciences, Division of Pharmaceutical Technology, University of Basel, Klingelbergstrasse 50 CH-4056 Basel, Switzerland

[a]Both authors contributed equally to this work.

Corresponding authors: *Cornelia.Palivan@unibas.ch
*Wolfgang.Meier@unibas.ch

Supplementary Materials

All materials were purchased from Sigma Aldrich unless otherwise stated.

Supplementary Methods

Confocal laser Scanning microscopy (CLSM)

CLSM measurements were performed using a confocal laser scanning microscope (ZEISS LSM 880, inverted microscope ZEISS Axio Observer, Carl Zeiss, Germany) equipped with a water immersion objective (C-Apochromate 40x/1.2W korr FCS M27) using the Zen Black software. Samples containing GFP and Q-dots were excited with an argon laser at 488 nm by setting the detector range to 499-521 nm for GFP and 640-714 for Q-dots. To measure the fluorescence of RFP-actin, SRB, resorufin-like product, and RFP, a diode-pumped solid-state 561-10 laser (DPSS) at 561 nm was used as excitation source and the detector range set to 572-704 nm. A helium-neon laser (HeNe) at 633 nm was used to measure samples containing CellTracker® Deep Red, and the fluorescence signal was collected with a detector range of 651-740 nm. Respectively, nano-compartment beam splitters (MBS) of 488, 561 and 633 were used. Each sample was scanned unidirectional using 1024 x 1024 pixels with a Bit Depth of 16 Bit. Afterwards, every raw image was adjusted with ImageJ software. 3D reconstructions and particle tracking analysis were done using Imaris software.

Cells were imaged either 24 h after treatment with transfection reagents or nano-formulations. Cells stained with CellTracker® Deep Red were imaged directly after the final washing step. To minimize spectral crosstalk the samples were scanned using the sequential mode. The laser settings including photomultiplier tube gain and pinhole settings were kept constant during the analyses between cells that were untreated and cells treated by transfection or addition of dye/nano-formulation.

GPMV Formation

GPMV formation was induced as described below by using a chemical “vesiculation” buffer 25 mM PFA/2 mM DTT in 10 mM HEPES, 150 mM NaCl, 2 mM CaCl2, pH7.4).

Isolation of GMPVs, E-GPMVs and MFs

The supernatant (600 µL) from 6-well cell culture plates (9 cm^2) was transferred to an Eppendorf tube and centrifuged at 100 g for 10 min to remove cells. 200 µL of the supernatant was transferred to poly-D-lysine- or plasma-activated 8-well microscopy chamber (Nunc™ Lab-Tek™ Chamber Slide System, Thermo Fisher Scientific).

CellTracker™ Deep Red staining and GPMV packaging

HepG2 cells were either cultured at a density of $4x10^4$ cells per well in a µ-Slide 8 well, Collagen IV: coverslip, or at a density of $30x10^4$ in a 6 well, in 200 µL or 500 µL DMEM growth medium (High Glucose Dulbecco’s Modified Eagle Medium (DMEM), supplemented

with 10% fetal calf serum, penicillin (100 units mL^{-1}) and streptomycin (100 µg mL^{-1})). 1 min after culturing the cells 10 µL of CellLight® Plasma Membrane-GFP BacMam 2.0 was added for cells cultured in 8 wells and 25 µL for cells cultured in 6 wells. This protocol was adapted in order to increase the total surface area of cells to which the transfection reagent can bind. Subsequently, cells were incubated for 24 h at 37ºC, 5% CO_2 to allow attachment on the surface. After 24 h the medium was removed and CellTracker® Deep Red was added according to the manufacturer's instructions. Cells were incubated for 30 min, and then washed five times with DMEM growth medium before inducing the vesiculation by adding the GPMV formation buffer.

E-GPMVs membrane modifications PEG5000, DiI,

HepG2 cells were either cultured at a density of $4x10^4$ cells per well in a µ-Slide 8 well, Collagen IV: coverslip or at a density of $30x10^4$ in a 6 well, in 200 µL or 500 µL DMEM growth medium (High Glucose DMEM, supplemented with 10% fetal calf serum, penicillin (100 units mL^{-1}), and streptomycin (100 µg mL^{-1})). Cells were incubated for 24 h at 37ºC, 5% CO_2 to allow attachment to the surface.
For Chol-PEG5000 membrane modifications, Chol-PEG5000-FITC, Chol-PEG5000-CLS, and Chol-PEG5000-Cy5 were purchased from Nanocs Inc. and a 20 µM solution was prepared in DMEM growth medium[1]. Cells were treated with Chol-PEG-5000-FITC or Chol-PEG5000-CLS for 10 min before removing the medium, washing the cells with DMEM and adding GPMV formation buffer. For *in vivo* experiments cells were treated with a Chol-PEG-5000-FITC and Chol-PEG-CLS 20 µM solution in DMEM for 1 h before removing the medium, washing the cells with PBS and adding the GPMV formation buffer.
For staining purposes, cells were treated with Chol-PEG-5000-FITC and Chol-PEG-Cy5 20 µM solution for 1 minute, before removing the medium, washing the cells with PBS and adding

Asialoglycoprotein receptor antibody staining

HepG2 cells were cultured at a density of $30x10^4$ in a 6 well, in 500 µL DMEM growth medium (High Glucose DMEM, supplemented with 10% fetal calf serum, penicillin (100 units mL^{-1}), and streptomycin (100 µg mL^{-1})). Cells were incubated for 24 h at 37ºC, 5% CO_2 to allow attachment to the surface. GPMV formation was induced by washing the cells with DMEM growth medium and adding the GPMV formation buffer.
5h after formation 200 uL of the GPMV solution was mixed with 1uL of the polyclonal Asialoglycoprotein receptor 1 antibody (HPA011954; Prestige Antibodies®, Sigma-Aldrich, St Louis, MO, USA), followed by a 10 minute incubation with a fluorescent-labelled secondary antibody (Atto-488). Unmodified GPMVs served as control. GPMVs were analyzed by flow cytometry, and at least 3000 events were collected.

GFP and RFP transfection of HepG2 cells

Cells were transfected using the mammalian expression plasmids pTagGFP-N and pTagRFP-N (Evrogen) encoding for the enhanced GFP or RFP under control of the cytomegalovirus promotor. Cells were seeded at a density of 2.5 x 10^4 cells/cm^2 on poly-D-lysine-coated or plasma-treated tissue culture slides, and allowed to adhere for 24 h. For the transfection, plasmid DNA (pDNA) was complexed using Lipofectamine 3000 at a pDNA-to-Lipofectamine reagents ratio (w/V) of 2. Cells were transfected using 2.5 µg pDNA per 1000 mm^2 surface area. GPMV formation was induced 24 h post transfection.

GFP and RFP GPMV packaging

HepG2 cells were either cultured at a density of 4x10^4 cells per well on a µ-slide 8-well, Collagen IV: coverslip, or at a density of 30x10^4 on a 6-well, in 200 µL or 500 µL DMEM growth medium, and streptomycin (100 µg mL^{-1})), respectively. Cells were incubated for 24 h at 37ºC, 5% CO_2 to allow attachment to the surface. After 24 h, cells were genetically modified as described to express GFP, RFP or both (co-transfection of GFP and RFP encoding plasmids). After 24 h, the medium was removed and GPMV formation buffer was added. Cells were imaged after incubation with GPMV formation buffer for 1 h at 37ºC, 5% CO_2.

RFP actin transfection and GPMV packaging

HepG2 cells were cultured as described above. Directly after cell seeding, 10 µL of CellLight® Plasma Membrane-GFP BacMam 2.0 and CellLight® Actin-RFP, BacMam 2.0[2] transfection reagents were added to cells cultured in 8 wells and 25 µL for cells cultured in 6 wells, respectively. After 24 h the medium was removed and GPMV formation buffer was added. Cells were imaged after incubation with GPMV formation buffer for 1 hour at 37ºC, 5% CO_2.

Flow cytometry analysis

Flow cytometry analysis was performed using a BD FACSCanto II flow cytometer (BD Bioscience, USA). Doublets were excluded using FSC-A and FSC-H detectors. For E-GPMVs modifications – single giants were excited at 488 nm or 640 nm, and the emission was detected using either the FL-1 (533/30; FITC Channel) or FL-3 (670; CTDR Channel). A minimum of 3000 events was recorded for each sample (n=3) were analyzed, and data processed using Flow Jo VX software (TreeStar). The samples were measured in "slow" flowthrough mode with the cytometer noozle kept at an appropriate distance from the sampling tube in order to prevent shear forces, which could influence the GPMV integrity. Size was calibrated using Sphero™ Particle size standards (Spherotech).

Liposome preparation

Liposomes were prepared using the film-rehydration-extrusion method. In brief, dry lipid films were rehydrated at 10ºC above the melting temperature, T_m, using a 1 mM sulforhodamine B solution, and resulting in a total lipid concentration of 40 mM. Finally, samples were extruded 21 times through a 100 nm diameter pore-size polycarbonate membrane at T_m+10ºC using an Avanti mini-extruder (Avanti Polar Lipids, Alabama, USA).
Non-encapsulated dye was removed from the liposomes by using a HiTrap Desalting ready-to-use 5 mL column, prepacked with Sephadex G-25 Superfine, equilibrated with PBS.
The following lipid formulations were prepared: DSPC:chol:DSPE-PEG (48:42:10 mol%) and DSPS:chol:DSPE-PEG (53:42:5 mol%).

DSPS and DSPC liposome uptake and packaging

HepG2 cells were cultured as described above. Directly after cell seeding, 10 µL of CellLight® Plasma Membrane-GFP BacMam 2.0 transfection reagent was added for cells cultured in 8 wells and 25 µL for cells cultured in 6 wells. Cells were incubated for 24 h at 37ºC, 5% CO_2 to allow attachment on surface. After 24 h the medium was removed and DSPS-based liposomes or PEGylated DSPC-based liposomes were added to a final total lipid concentration of 1mg mL^{-1}. Cells were incubated for an additional 24 h in DMEM growth medium. After 24 h cells were washed five times with DMEM growth medium. After the final wash step, DMEM growth medium was removed and GPMV formation buffer was added. Cells were imaged after incubation with GPMV formation buffer for 1 h at 37ºC, 5% CO_2.

Preparation of SRB-loaded PMOXA-PDMS-PMOXA polymersomes

SRB-loaded polymersomes were prepared at RT from the ABA triblock copolymer, $PMOXA_6$-$PDMS_{44}$-PMOXA6. Polymersomes were generated using the film rehydration technique in which the polymer was dried, and 1 mM sulforhodamine B in PBS buffer (pH=7.4) at RT was then used as a rehydration solution. Rehydrated films were stirred overnight at RT, while being protected from light. All samples were extruded through an Avanti mini-extruder (Avanti Polar Lipids, USA) using a 100 nm diameter pore-size polycarbonate membrane (7 times) at RT in order to obtain size homogeneity. Non-encapsulated dye was removed from the polymersomes by using a HiTrap Desalting ready-to-use 5 mL column, pre-packed with Sephadex G-25 Superfine, and equilibrated with PBS.

PMOXA-PDMS-PMOXA polymersome uptake and E-GPMVs packaging

HepG2 cells were cultured as described above. Directly after cell seeding, 10 µL of CellLight® Plasma Membrane-GFP BacMam 2.0 transfection reagent was added for cells cultured in 8 wells and 25 µL for cells cultured in 6 wells. Cells were incubated for 24 h at 37ºC, 5% CO_2 to allow attachment on surface. After 24h the medium was removed and a solution of PMOXA-PDMS-PMOXA polymersomes was added to a final concentration of 0.5mg mL^{-1}, 1mg mL^{-1}, 2mg mL^{-1} and 3mg mL^{-1}. After 24 h, cells were washed five times with DMEM growth medium. After the final wash step, DMEM growth medium was removed and GPMV formation buffer was added. Cells were imaged after incubation with GPMV formation buffer for 1 h at 37ºC, 5% CO_2.

Preparation of PMOXA-PDMS-PMOXA based AOs

AOs (OmpF equipped polymersomes loaded with HRP) were prepared at RT from the ABA triblock copolymer, $PMOXA_6$-$PDMS_{44}$-$PMOXA_6$, ($A_6B_{44}A_6$), OmpF and Horseradish peroxidase (HRP) as described in Einfalt et al 2018[3]. The synthetic procedure and the polymer characterization are presented in Itel et al 2017[4]. In brief, polymersomes were generated using the film rehydration technique where the polymer was dried in the presence of OmpF K89 R270 cysteine mutant. Horseradish peroxidase type IV (Sigma Aldrich) in PBS buffer pH=7.4 at RT were used as rehydration solutions. Films were rehydrated with PBS pH 7.4 to a final polymer concentration of 2.5 mg mL^{-1}, HRP concentration of 0.2 mg mL^{-1}, and OmpF concentration of 100 µg mL^{-1}, respectively. Control polymersomes were also prepared in the absence of OmpF. Rehydrated films were stirred in the dark overnight at RT. All samples were extruded through an Avanti mini-extruder (Avanti Polar Lipids, USA) using a 100 nm diameter pore-size polycarbonate membrane (11 times) at RT in order to obtain size homogeneity. Non-encapsulated enzyme was removed from the polymersomes by dialysis against PBS at pH 7.4 at RT for 4 days using Spectrapore dialysis tubes, MWCO 300 kDa (Spectrum Laboratories Inc., USA); the buffer was exchanged 3x in 4 hour intervals during

the day, but was left unchanged overnight. Steps involving the generation of catalytic nano-compartments were performed in the dark in order to avoid photo degradation of fluorophores. Activity measurements were performed on the day following the last dialysis step.

Characterization of AOs

The reaction of HRP-loaded nano-compartments equipped with OmpF or lacking OmpF was studied employing the Amplex UltraRed enzymatic assay. Substrate conversion was determined by observing the increase in fluorescence intensity (λ_{Ex}:530 λ_{Em}:590 nm) in time. The emission fluorescence intensity was determined using a LS 55 Fluorescence Spectrometer (Perkin Elmer). Samples were incubated with a final concentration of 0.05 mg mL^{-1} AOs in PBS at pH 7.4, 4.5 µM H_20_2 and 3.4 µM Amplex UltraRed. Fluorescence was expressed as relative fluorescence units (RFU) and was measured at the same instrument setting in all experiments.

Characterization of *in vitro* activity of MFs

The reaction of E-GPMVs loaded with catalytic HRP-loaded polymersomes equipped with OmpF or lacking OmpF was studied employing the Amplex UltraRed enzymatic assay using the microscrope set-up described above. Substrate conversion was determined by observing the fluorescence intensity by CLSM using a diode-pumped solid-state 561-10 laser (DPSS) at 561 nm as excitation source and the detector range set to 572-704 nm. Formed MFs, were incubated with a final concentration of 2.25 µM H_2O_2 and 1.7 µM Amplex UltraRed for 2 h after GPMV formation. Subsequently, the reaction was visualized using a continuous recording.

MF preparation for ZFE injection.

HepG2 cells were cultured at a density of $30x10^4$ in a collagen coated 6 well in 500 µL DMEM growth medium. Cells were incubated for 24 h at 37ºC, 5% CO_2 to allow attachment to the surface.

In the first set-up of injected E-GPMVs, RFP transfection agent was added post cell attachment, as described above. Subsequently 24 h after RFP transfection cells were treated with 20 µM Cholesterol-PEG5000 solution in PBS for 30 min, the cells were washed with cell-culture medium, the medium was removed and GPMV formation buffer was added.

In the second set up, cells were incubated with CTDR according to the manufacturer’s instructions. CTDR loaded cells were washed with culture medium 30 min after incubation with CTDR and treated with 20 µM Cholesterol-PEG5000-FITC for additional 30 min. Cells were washed with GPMV formation buffer, the medium was removed and GPMV formation buffer was added.

In the final set-up, cells were incubated with HRP-loaded PMOXA-PDMS-PMOXA polymersomes equipped with OmpF or lacking OmpF (AOs) at a final concentration of 1 mg mL^{-1} for an additional 24 h after their attachment to the surface. Subsequently 24 h post incubation with AOs, cells were washed extensively and treated with 20 µM Cholesterol-PEG5000-FITC for additional 30 min. Cells were washed with GPMV formation buffer, the medium was removed and GPMV formation buffer was added. GPMVs were collected from the cells as described above, sedimented overnight and injected into ZFE on the following day.

Zebrafish injections

Standard zebrafish embryo (ZFE) culture medium at pH 7.4 was prepared at final concentrations of 5 mM sodium chloride, 0.25 mM potassium chloride, 0.5 mM magnesium sulfate, 0.15 mM potassium dihydrogen phosphate, 0.05 mM sodium phosphate dibasic, 0.5 mM calcium chloride, 0.71 mM sodium bicarbonate, and 0.001 % (w/v) methylene blue. Collected eggs from adult ABC/TU ZFE (wildtype) and kdrl:EGFP (GFP marked vasculature) were kept in ZFE culture medium at 28°C. PTU (0.03 mg mL^{-1}) was added 1-day post fertilization (dpf) in order to avoid pigment cell formation. E-GPMVs were injected into anesthetized (0.01% tricaine (w/v)) 2-dpf ZFE according to an adapted protocol originally designed for microangiography or 4-dpf into the peritoneal cavity. ZFE were cast into 0.3% (w/v) agarose containing the same amount of tricaine. Immobilized ZFE were injected with either with 3 nL of E-GPMVs solution or approximately 3nL of MF solution, pre-incubated at a final concentration of 5 µM H_20_2 and 3.4 µM Amplex UltraRed for 2 h. Directly after the injection of MFs, 3 nL of 110 µM and 75 µM Amplex UltraRed were injected in the same location in order to further increase the substrate concentrations in ZFE.

Characterization of *in vivo* activity of MFs

Activity of MFs loaded with catalytic HRP-loaded polymersomes equipped with OmpF or lacking OmpF *in vivo* was studied employing the Amplex UltraRed enzymatic assay using the microscrope set-up described above. Substrate conversion was determined by observing the fluorescence intensity by CLSM using a diode-pumped solid-state 561-10 laser (DPSS) at 561 nm as excitation source and the detector range set to 572-704 nm. Subsequently, the reaction was visualized using a continuous recording.

Colocalization analysis of fluorescent signals in GPMVs

Colocalization analysis of different fluorescent markers inside GPMVs was carried out using the JaCoP plug-in in the Fiji software. Both Pearson's Correlation Coefficient (PCC) and Manders' Correlation Coefficients M1/M2 (automatic threshold) were used to quantify the degree of colocalization (considering different fluorescence intensity effects). Coefficients close to 1 indicate a high degree of colocalization, whereas coefficients close to 0 indicate fluorescent signals which do not overlap[3,5].

Transmission electron microscopy (TEM)

4 mM total lipid concentration of DSPS:Cholesterol:DSPE-PEG (53:42:5 %mol) liposomes, 4 mM total lipid concentration of DSPC:Cholesterol:DSPE-PEG (48:42:10 %mol) liposomes, 0.1mg mL^{-1} total polymer concentration $PMOXA_{44}$-$PDMS_6$-$PMOXA_{44}$ polymersomes, 0.5 mg mL^{-1}, HRP-loaded polymersomes or HRP-loaded polymersomes equipped with OmpF solution were used for TEM imaging studies. Samples were negatively stained with 1.5% uranyl acetate solution and deposited on plasma activated carbon-coated copper grids. A transmission electron microscope (Philips Morgagni 268D) was used.

Dynamic light scattering

The measurements of D_H of nano-formulations were performed on a Zetasizer Nano ZSP at 25°C. The measuring angle was 173° and the data were analyzed by intensity distribution using a sample volume of 450 µL.

Fluorescence correlation spectroscopy (FCS)

All FCS measurements were performed using a confocal laser scanning microscope (ZEISS LSM 880, inverted microscope ZEISS Axio Observer, Carl Zeiss, Germany) equipped with a water immersion objective (C-Apochromate 40x/1.2W korr FCS M27). Measurements were done at RT using a sample volume of 20 µL calibration solutions (SRB, nano-encapsulated SRB liposome or polymersome solutions) and 200 µL for GPMV solutions on a covered eight-well Lab-Tek chambered borosilicate cover glass (Nalage Nunc International, USA). A diode-pumped solid-state 561-10 laser (DPSS) at 561 nm was used as excitation source for the excitation of Sulforhodamine B along with the appropriate filter sets. The fluorescence signal was measured in real-time and the autocorrelation function was calculated by a software correlator. Measurements were recorded over 2 s and each measurement was repeated 60 times.
For calibration R and τ_D of free SRB were determined independently in PBS pH 7.4. Experimental auto correlation curves of the free fluorophore were fitted using a one component model including the triplet state (Supplementary Equation 1):

$$G(\tau)_{fit} = 1 + (1 + \frac{T}{1-T} e^{-\frac{\tau}{\tau_{trip}}}) \frac{1}{N} \left[\frac{1}{1+\frac{\tau}{\tau_D}} \frac{1}{\sqrt{1+R^2\frac{\tau}{\tau_D}}} \right]$$

τ_D represents the diffusion time, T the fraction of fluorophores in the triplet state with triplet time τ_{trip}, N is the number of particles and R the structural parameter. Measurements were exported from the Zen, ZEISS LSM 880 software (Black edition) and median values determined using GraphPad Prism 6.0, based on individual FCS measurements (n=60).

For determining the free dye fraction of SRB in purified SRB-loaded liposomes/polymersomes and polymersomes compartmentalized within GPMVs, experimental auto correlation curves were fitted using a two-component model including a triplet state (Supplementary Equation 2):

$$G(\tau)_{fit} = 1 + \left(1 + \frac{T}{1-T} e^{-\frac{\tau}{\tau_{trip}}}\right) \frac{1}{N} \left[\frac{f_1}{1+\frac{\tau}{\tau_{D1}}} \frac{1}{\sqrt{1+R^2\frac{\tau}{\tau_{D1}}}} \right] + \left(1 + \frac{T}{1-T} e^{-\frac{\tau}{\tau_{trip}}}\right) \frac{1}{N} \left[\frac{f_2}{1+\frac{\tau}{\tau_{D2}}} \frac{1}{\sqrt{1+R^2\frac{\tau}{\tau_{D2}}}} \right]$$

τ_D of free dye (Sulforhodamine B) was determined independently in PBS pH 7.4, and subsequently fixed along with R (5) during the two component fitting procedure.
For intra GPMV measurements single GPMVs were selected based on CLSM micrographs using the position tool. Correlation curves, which presented molecular brightness values 10% below the free fluorophore (CPM = 13 kHz) or presented diffusion times above 25 0000 µs were excluded from analysis (<30%).

Cryogenic-TEM

$PMOXA_6$-$PDMS_{44}$-$PMOXA_6$ polymersome suspensions in buffer (10 mM PBS, pH 7.4, 50 mM NaCl) at high concentrations (2.5 mg mL^{-1}) were deposited on glow-discharged carbon grids (Quantifoil, Germany) and blotted before quick-freezing in liquid ethane using a Vitribot plunge-freezing device (FEI Co.). The grids were stored in liquid nitrogen before transferring them into a cryo-holder (Gatan). Imaging was performed on a Philips CM200

FEG TEM at 200 kV with accelerating voltage in low-dose mode and a defocus value of about -4 µm.

OmpF expression and extraction

OmpF extraction procedure was adapted from Einfalt et al. 2018[3]. In brief, under sterile conditions, cells of an ampicillin resistant Escherichia Coli (E. Coli) strain BL21 stock culture overexpressing the cysteine OmpF K89 R270 mutant, previously described in Edlinger et al. 2017 [6] were smeared onto the surface of LB, ampicillin agar plate (100 mg mL^{-1}). After 16h incubation at 37 °C, a single bacterial colony was picked and transferred from the ampicillin agar plate and transferred to 15 mL^{-1} of TB liquid medium with 100 mg mL^{-1}ampicillin (total 5 flasks). 3 times 1 L of TB medium with 100 mg mL^{-1}ampicillin was inoculated with 10 mL of the overnight culture. The optical density (OD) of the growing *E. Coli* culture was followed by measuring the absorbance at 600 nm using Nanodrop. Once the absorbance reached an OD of 0.6, Izopropil-β-D-1-tiogalaktopiranozid (IPTG) was added to a final concentration of 1 mM into each flask in order to start the expression of the OmpF K89 R270 gene. Bacteria were grown for 16 more hours at 25ºC with vigorous shaking. Then the *E. Coli* cells were pelleted at 9.000 x g for 10 min at 4ºC. Pellets were stored at -8ºC, and on the day following the harvesting re-suspended in a total of 50 mL of 25 mM Tris-HCl at pH 7.4. 10 mg DNAse and 10 mg RNAse were added after the cell suspension was French pressed five times at 1,000 bar. 1 h after incubation with DNAse and RNAse, 1 mL of 20% SDS was added per 10 mL of cell lysate and incubated for 1 h at 60ºC. The suspension was centrifuged at 36.000 x g at RT. The supernatant was removed and the cell pellet incubated in 0.125% n-octyl-β-D-glucopyranoside (OG) for 1 h at 37ºC. The suspension was centrifuged at 36.000 x g at RT. Finally, the cell pellet was re-suspended in 3% OG (Anatrace, USA) in 10 mM phosphate buffer and homogenized. The final suspension was centrifuged at 50.000 x g and the protein concentration in the supernatant were determined by UV-Visible absorption spectroscopy at 280 nm using Nanodrop (Thermofischer Scientific, Switzerland).

HRP and OmpF Characterization

A 4-15% Mini-PROTEAN® TGX™ Precast SDS (Bio-Rad Laboratories, USA) gel polyacrylamide gel was used, then samples were mixed with Laemmni loading buffer and 15 µL of the OmpF solution was added to the gel. SDS-PAGE gels were run at 200 V for 45 min and scanned stained with Simply Blue (Thermofischer).

Photobleaching experiments

All photo bleaching experiments were carried out using a confocal laser scanning microscope (ZEISS LSM 880, inverted microscope ZEISS Axio Observer, Carl Zeiss, Germany) equipped with a water immersion objective (C-Apochromate 40x/1.2W korr FCS M27). Photo bleaching experiments were done at RT using FCS. Single RFP GPMVs or ZFE melancoytes were selected using the position mode in FCS. A diode-pumped solid-state 561-10 laser (DPSS) at 561 nm was used as excitation source for the excitation and subsequent bleaching of RFP along with the appropriate filter sets. The fluorescence signal was measured in real-time. Single measurements were recorded over 1 s and each measurement was repeated 60 times for a selected GPMV.

Statistical analysis

A. Statistical analysis of the GPMV sizes
The empirical distribution parameters for the GPMVs without Chol-PEG5000 and with Chol-PEG5000 are:

GPMVs without Chol-PEG5000:
min: 1527 max: 24248
median: 7012.5
mean: 7739.412
estimated sd: 3702.318
estimated skewness: 1.079705
estimated kurtosis: 4.113617

GPMVs with Chol-PEG5000:
min: 1375 max: 19937
median: 7623
mean: 8509.361
estimated sd: 4085.349
estimated skewness: 0.6342648
estimated kurtosis: 2.572434

The GPMVs size distribution has been fitted with a lognormal distribution function.

GPMVs without Chol-PEG5000:

Fitting of the distribution ' lnorm ' by maximum likelihood
Parameters:
estimate Std. Error
meanlog 8.8448623 0.02110688
sdlog 0.4719642 0.01492452

GPMVs with Chol-PEG5000:
Fitting of the distribution ' lnorm ' by maximum likelihood
Parameters:

	estimate	Std. Error
meanlog	8.928874	0.02211693
sdlog	0.501913	0.01563875

Kolmogorov-Smirnov non-parametric test is used to verify if the lognormal distribution of GPMVs sizes is appropriate as statistical model.

GPMVs without Chol-PEG5000:
One-sample Kolmogorov-Smirnov test
data: gpmv1$NonPEG
D = 0.018222, p-value = 0.9964

GPMVs with Chol-PEG5000:

One-sample Kolmogorov-Smirnov test
data: gpmv2$PEG
D = 0.056507, p-value = 0.07459

The comparison of the mean values of the GPMV sizes without and with Chol-PEG5000:
Welch Two Sample t-test
data: gpmv1$NonPEG and gpmv2$PEG
t = -3.148, df = 1008.2, p-value = 0.001692
alternative hypothesis: true difference in means is not equal to 0
95 percent confidence interval:
-1249.9048 -289.9935
sample estimates:
mean of x mean of y
7739.412 8509.361

B. The analysis of the co-localization of red and green dyes when they are simultaneously present inside GPMVs, takes into account focal planes analyzed by CLSM. Error bars in the box plots correspond to a normal distribution of GPMVs from the same batch, and therefore represent the Q1, Q3 and the Q1 – 1.5 (IQR) and Q3 + 1.5(IQR). The sample number is indicated in the figure description as n.

C. OLS fit of the number of polymerosomes/GPMVs size for an initial concentration of polymersomes of 3 mg mL^{-1}.

```
Residuals:
    Min       1Q   Median       3Q      Max
-0.47846 -0.06504  0.02913  0.13348  0.33551

     Coefficients
EE         Estimate  Std. Error  t value  Pr(>|t|)
log(Size)  1.67308    0.19031    8.791   5.11e-06 ***
Size      -0.14228    0.04341  -3.278   0.00832 **
---
Signif. codes:  0 '***' 0.001 '**' 0.01 '*' 0.05 '.' 0.1 ' ' 1

Residual standard error: 0.2479 on 10 degrees of freedom
Multiple R-squared:  0.9893,    Adjusted R-squared:  0.9872
F-statistic: 464.3 on 2 and 10 DF,  p-value: 1.373e-10
```

Correlation test using Spearman's rank correlation rho:

```
data:  x and y
S = 62, p-value = 0.004115
alternative hypothesis: true rho is not equal to 0
sample estimates:
      rho
0.7832168
```

D. Distribution of the number of polymersomes/GPMV.

```
summary statistics
```

```
min:  0   max:  50
median:  6
mean:  8.693023
estimated sd:  8.587031
estimated skewness:  1.522656
estimated kurtosis:  5.992936
Fitting of the distribution ' geom ' by maximum likelihood
Parameters:
     estimate    Std. Error
      0.103167   0.006662553
```

Supplementary Figures

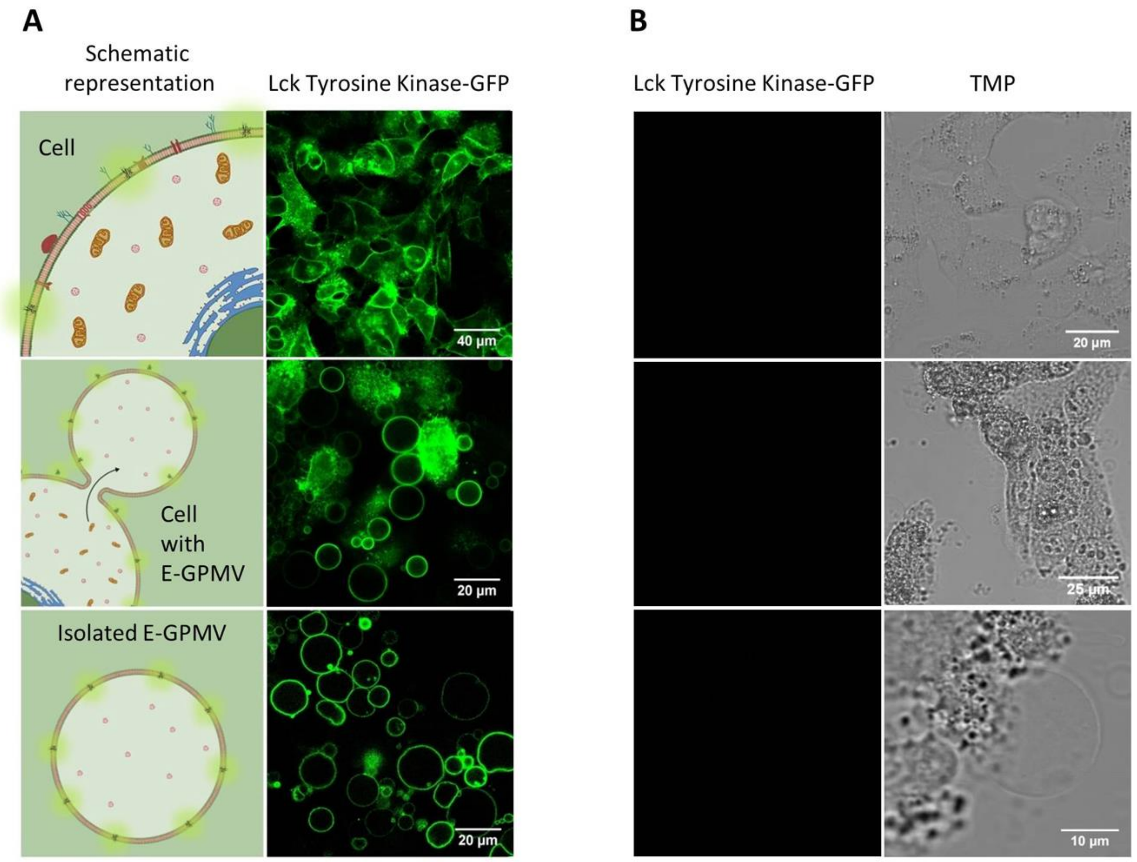

Figure S1. A.) Left column: Top) Schematic representation of HepG2 cells with enhanced membrane content. Central) Illustration of E-GPMV formation. Bottom: Finally, the isolated E-GPMV. Right column: CLSM micrograph of HepG2 cells containing the membrane protein Lck Tyrsoine Kinase-GFP (Green), followed by E-GPMV formation and finally the isolated E-GPMVs containing the transferred Lck Tyrosine Kinase-GFP in their membrane. B.) Control HepG2 cells, without Lck-Tyrosine Kinase-GFP modification, followed by HepG2 cells after GPMV formation.

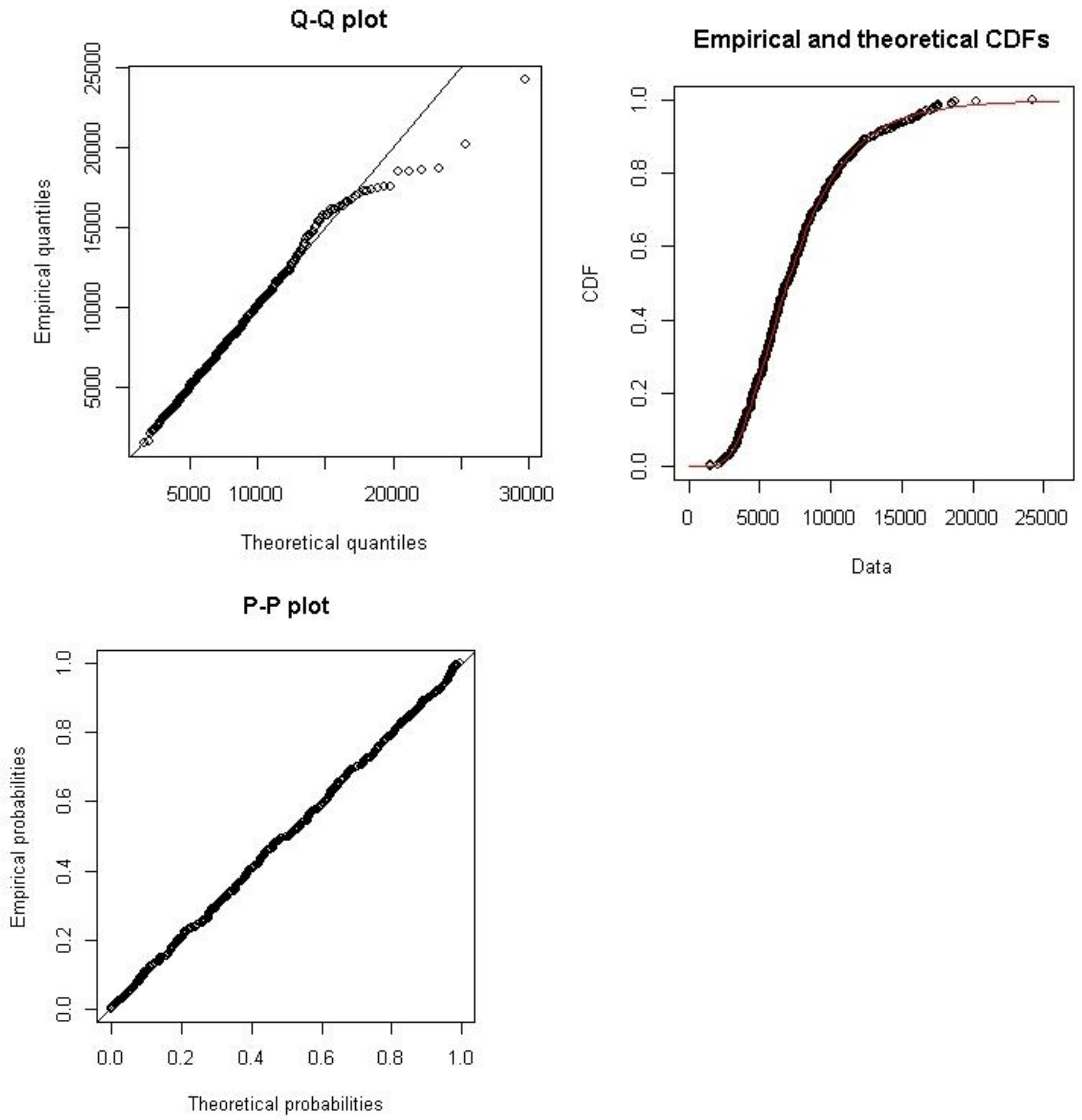

Figure S2. Statistical analysis of the distribution of the E-GPMV sizes obtained by CLSM. Q-Q plot of the empirical quantiles as function of the theoretical quantiles for the lognormal distribution. Cumulative distribution function of the empirical and lognormal distribution. P-P plot of the probabilities of the empirical and lognormal distribution.

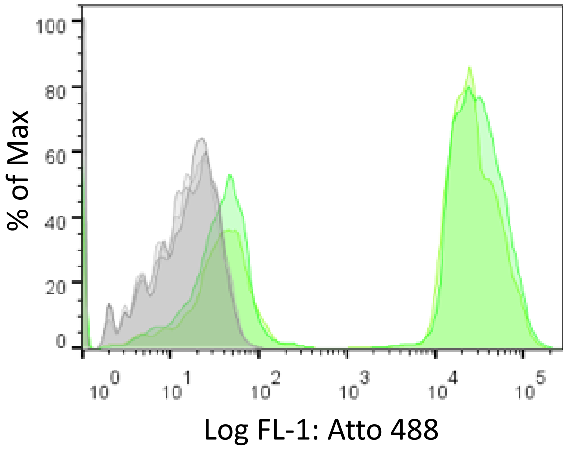

Figure S3. Flow cytometry analysis of HepG2 derived GPMVs. GPMVs stained with primary Anti-Asyaloglycoprotein receptor antibody and secondary Atto488 conjugated antibody (Green), and as control: unstained HepG2 derived GPMVs (Gray).

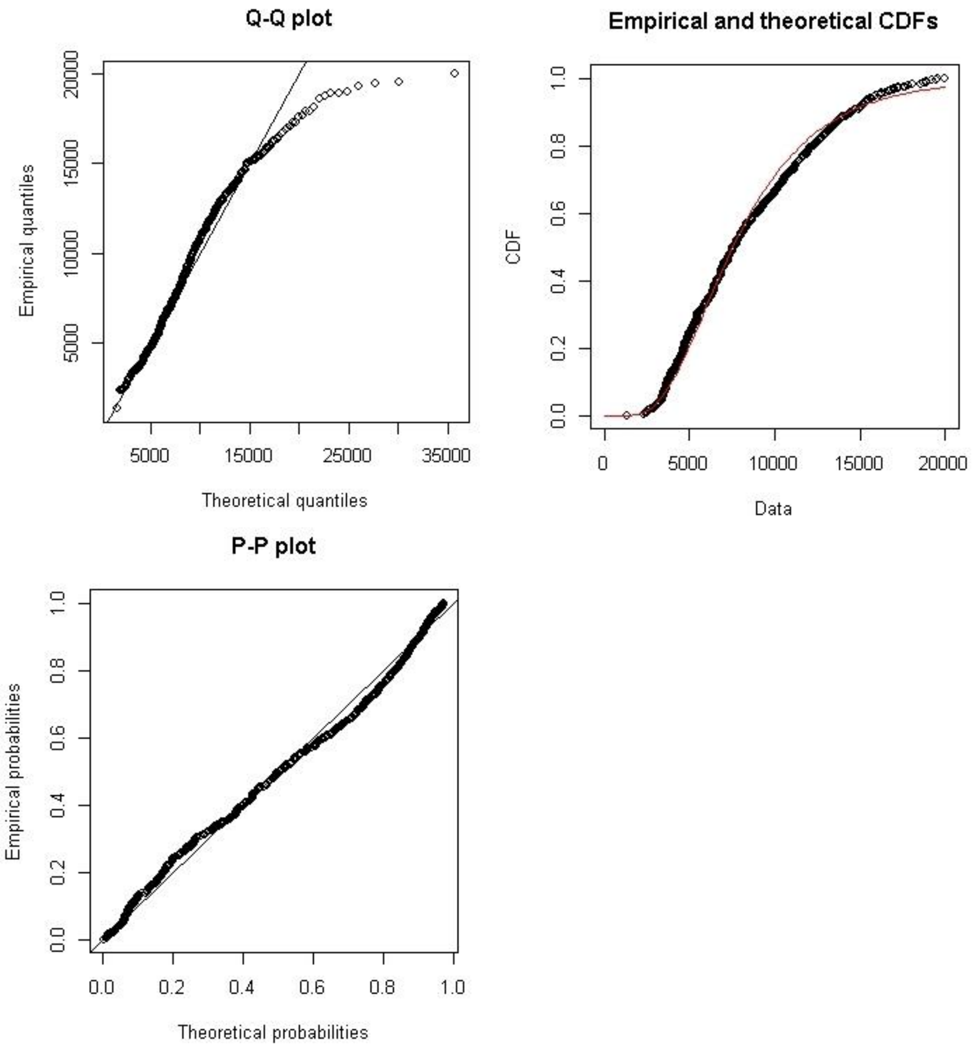

Figure S4. Statistical analysis of the distribution of the Chol-PEG5000-equipped E-GPMV sizes obtained by CLSM. Q-Q plot of the empirical quantiles as function of the theoretical quantiles for the lognormal distribution. Cumulative distribution function of the empirical and

lognormal distribution. P-P plot of the probabilities of the empirical and lognormal distribution.

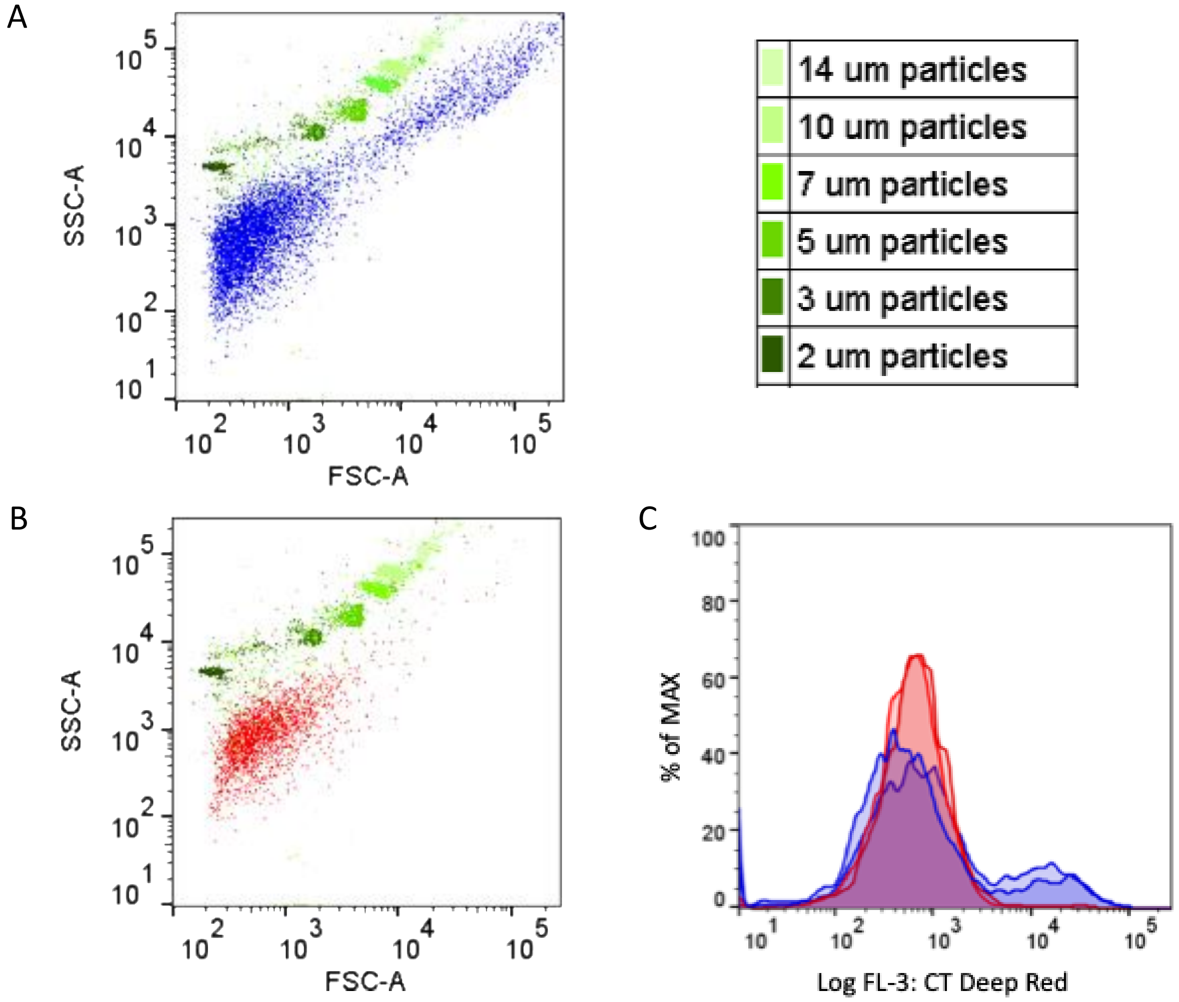

Figure S5. Flow cytometry analysis of size distribution of E-GPMVs with Chol-PEG5000 modified membranes (A) or native membranes (B), 24h post isolation. Size range color coded by polystyrene bead particles dark green to light green. C) Membrane leakiness of plasma membranes, measured by CTDR retention, measured by flow cytometry. CTDR loaded GPMVs equipped with native membranes (Red) and CTDR loaded E-GPMVs with Chol-PEG5000 modified membranes (Blue), 24h post isolation.

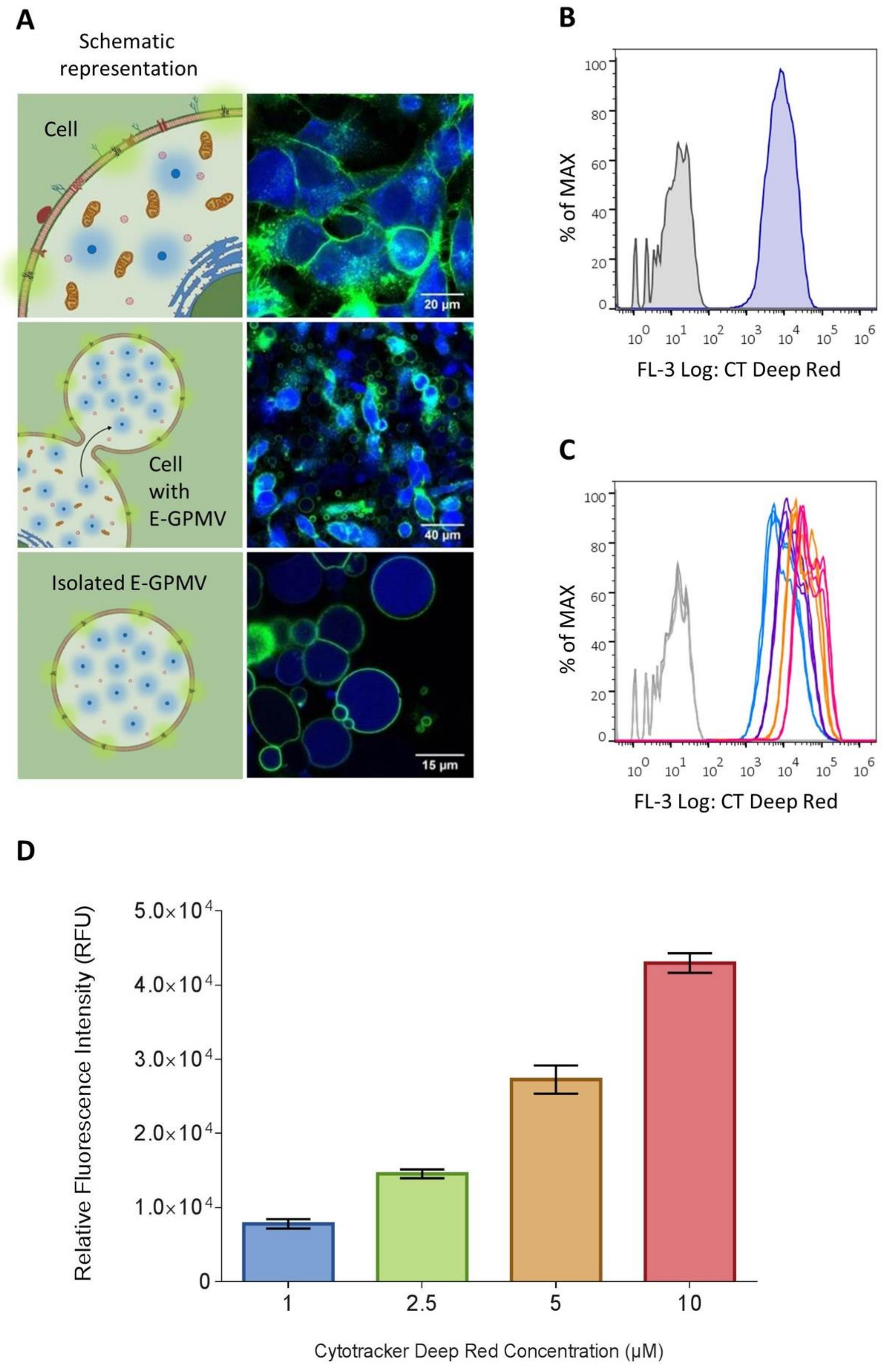

Figure S6. A) Left column: Top) Schematic representation of HepG2 cells with enhanced membrane and cytosolic content. Central) Illustration of E-GPMV formation. Bottom) Finally the isolated E-GPMV. Right column: CLSM micrograph of HepG2 cells containing the membrane protein Lck Tyrosine Kinase-GFP (Green) and the cytosolic CellTracker™ Deep Red dye (CTDR) (Blue), followed by E-GPMV formation and finally the isolated E-GPMVs

containing the transferred Lck Tyrsoine Kinase-GFP in their membrane and CTDR inside their cavity. B) Flow cytometry analysis of CTDR-loaded E-GPMVs (Blue) modified with Chol-PEG5000-FITC and control (unmodified, unloaded) GPMVs (Gray). C) Flow cytometry analysis of GPMVs derived from HepG2 cells cultured in the presence of 1 µM (Blue) 2.5 µM (Purple), 5 µM (Orange) and 10 µM (Red) CTDR [1,7]. D) CTDR transfer from cells to E-GPMVs. Fluorescence of E-GPMVs derived from cells cultured at different CTDR concentrations.

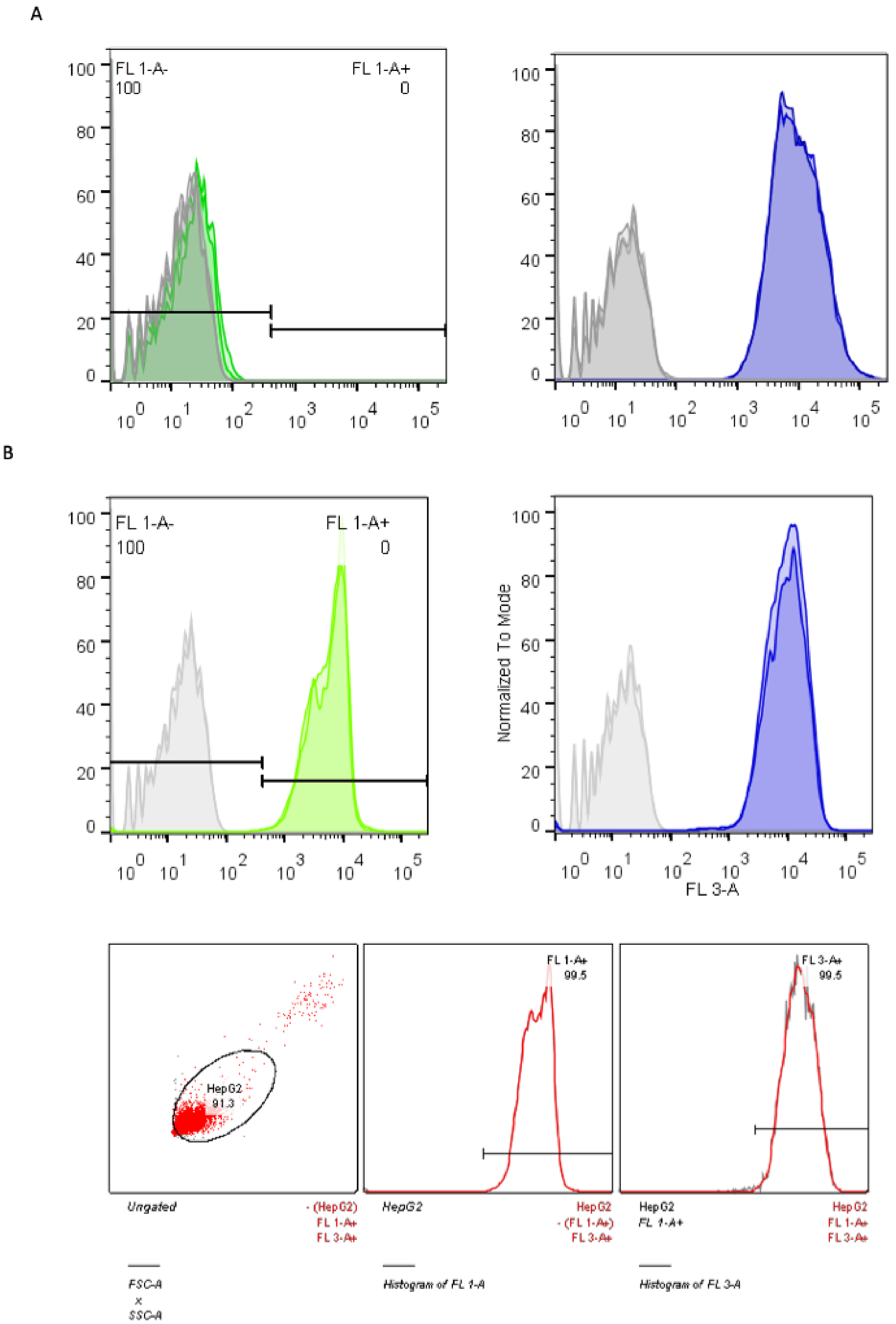

Figure S7. Back-gating flow cytometry analysis of E-GPMVs: A) CTDR loaded cavities and B) CTDR loaded cavities and Chol-PEG-5000-FITC equipped membranes. Unmodified GPMVs (Grey), Chol-PEG-FITC signal (Green) and CTDR signal (Blue).

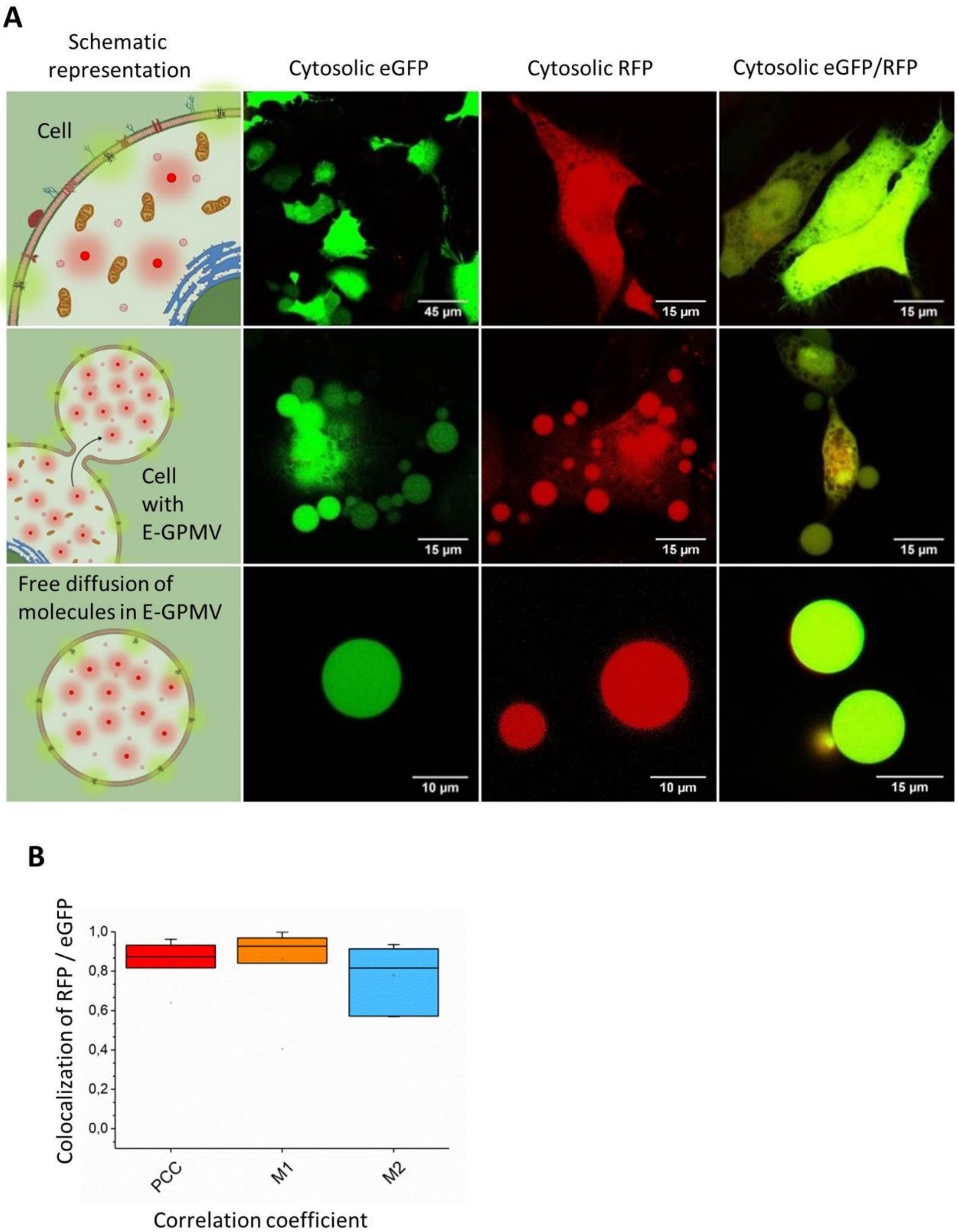

Figure S8. A) First column: Top) Schematic representation of HepG2 cells with enhanced cytosolic content. Central) Illustration of E-GPMV formation. Bottom) Finally the isolated E-GPMV. Second column: CLSM micrograph of HepG2 cells containing cytosolic eGFP (Green), followed by E-GPMV formation and finally the isolated E-GPMV containing the eGFP inside their cavity. Third column: Transfer of cytosolic RFP (Red) from the donor HepG2 cell to the isolated E-GPMVs. Forth column: Co-transfer of cytosolic eGFP and RFP (Yellow) from the donor HepG2 cell to the isolated E-GPMVs. B) Colocalization analysis of RFP and eGFP inside E-GPMVs. Pearson's Correlation Coefficient, PCC = 0.85 ± 0.11, Manders' Coefficient M1 = 0.86 ± 0.21, M2: Manders' Coefficient M2 = 0.78 ± 0.15. Median is based on n=7 independent CLSM images made from E-GPMVs from the same batch.

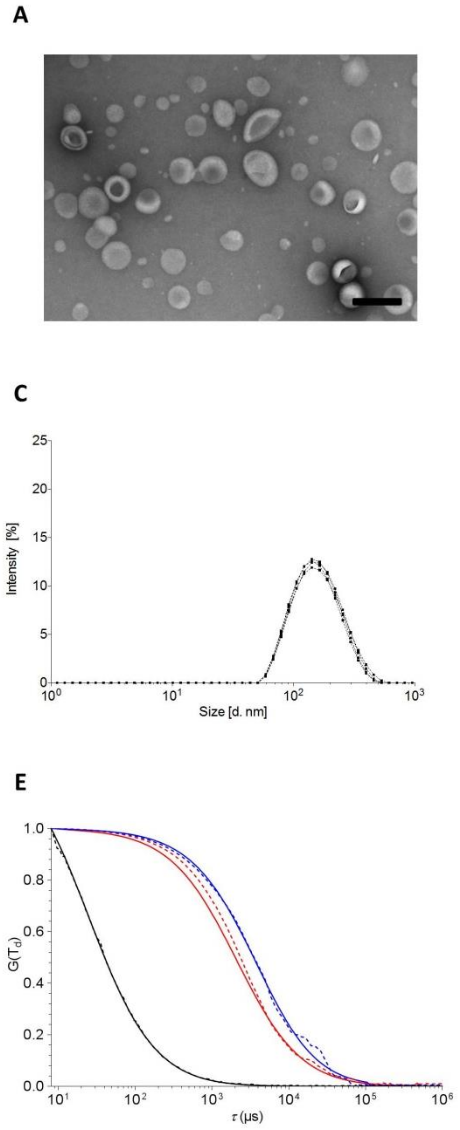

Figure S9. A) Transmission electron microscopy micrograph of SRB-loaded DSPC:Cholesterol:DSPE-PEG (48:42:10 %mol) liposomes in PBS pH 7.4. Scale bar represents 200 nm. B) Transmission electron microscopy micrograph of SRB-loaded DSPS:Cholesterol:DSPE-PEG (53:42:5 %mol) liposomes in PBS pH 7.4. Scale bar represents 200 nm. C) Intensity distribution by DLS of SRB-loaded DSPS:Cholesterol:DSPE-PEG (53:42:5 %mol) liposomes measured in PBS pH 7.4 (n=3). D) Intensity distribution by DLS of SRB-loaded DSPC:Cholesterol:DSPE-PEG (48:42:10 %mol) liposomes measured in PBS pH 7.4 (n=3). E) FCS analysis of SRB-loaded DSPS- and DSPC-based liposomes in PBS pH 7.4. FCS autocorrelation curves of free SRB in PBS pH 7.4 (Black), SRB-loaded DSPS-based liposomes in PBS pH 7.4 (Blue) SRB-loaded DSPC-based liposomes in PBS pH 7.4 (Red). Dotted line – experimental auto correlation curves, Full line – fit. Curves normalized to 1 to facilitate comparison.

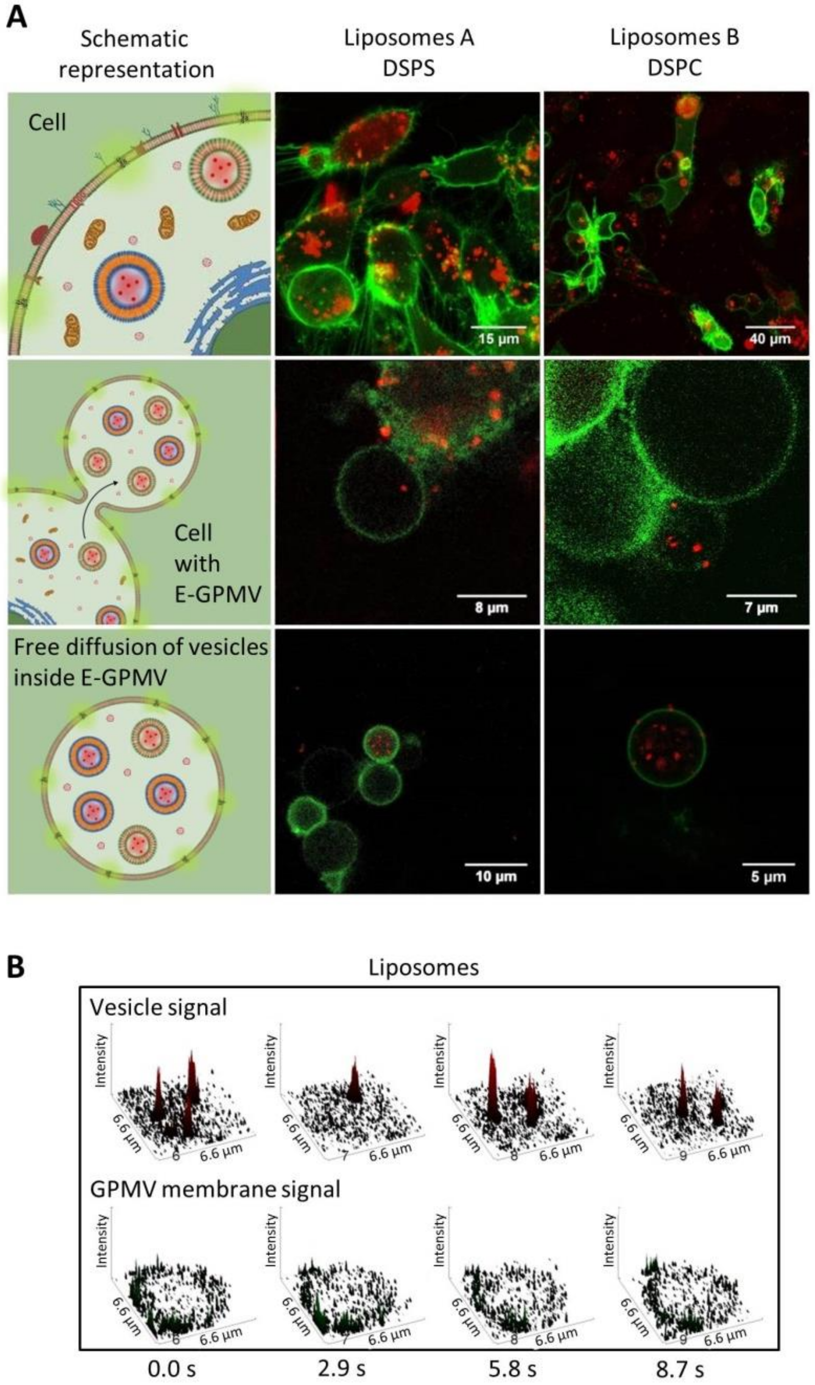

Figure S10. Subcompartmentalization within E-GPMVs. Panel A) Transfer of liposomes from the HepG2 cytoplasm into E-GPMV. Left column: Top) Schematic representation of HepG2 cells with enhanced membrane and cytosolic content. Central) Illustration of E-GPMV formation. Bottom) Finally the isolated E-GPMV. Middle column: CLSM micrograph of the co-transfer of LcK-GFP (green) and SRB-loaded DSPS-liposomes (Red) from the donor HepG2 cell to the isolated E-GPMV. Right column: CLSM micrograph of the co-transfer of LcK-GFP (green) and SRB-loaded DSPC-liposomes (Red) from the donor HepG2 cell to the isolated E-GPMV. Panel B) Single plane recordings of SRB-loaded DSPC-liposomes inside E-GPMVs measured by CLSM.
.

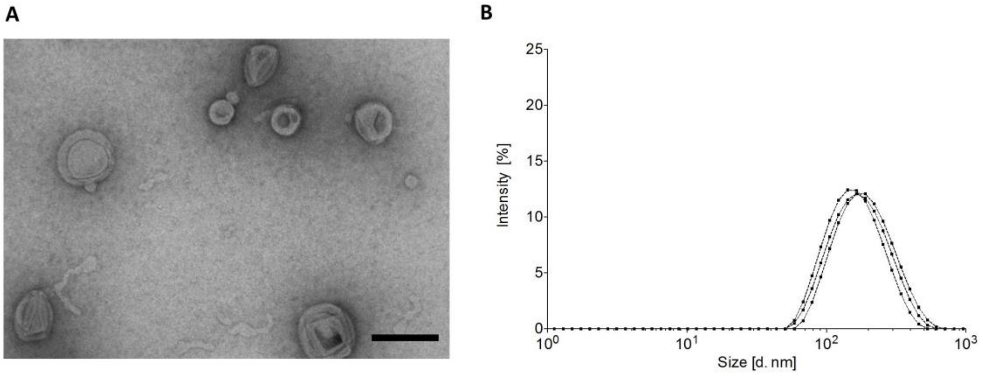

Figure S11. A) Transmission electron microscopy micrograph of SRB-loaded $PMOXA_6$-$PDMS_{44}$-$PMOXA_6$ polymersomes in PBS pH 7.4. Scale bar: 200 nm. B) Intensity distribution by DLS of SRB-loaded $PMOXA_6$-$PDMS_{44}$-$PMOXA_6$ polymersomes measured in PBS pH 7.4. (n=3).

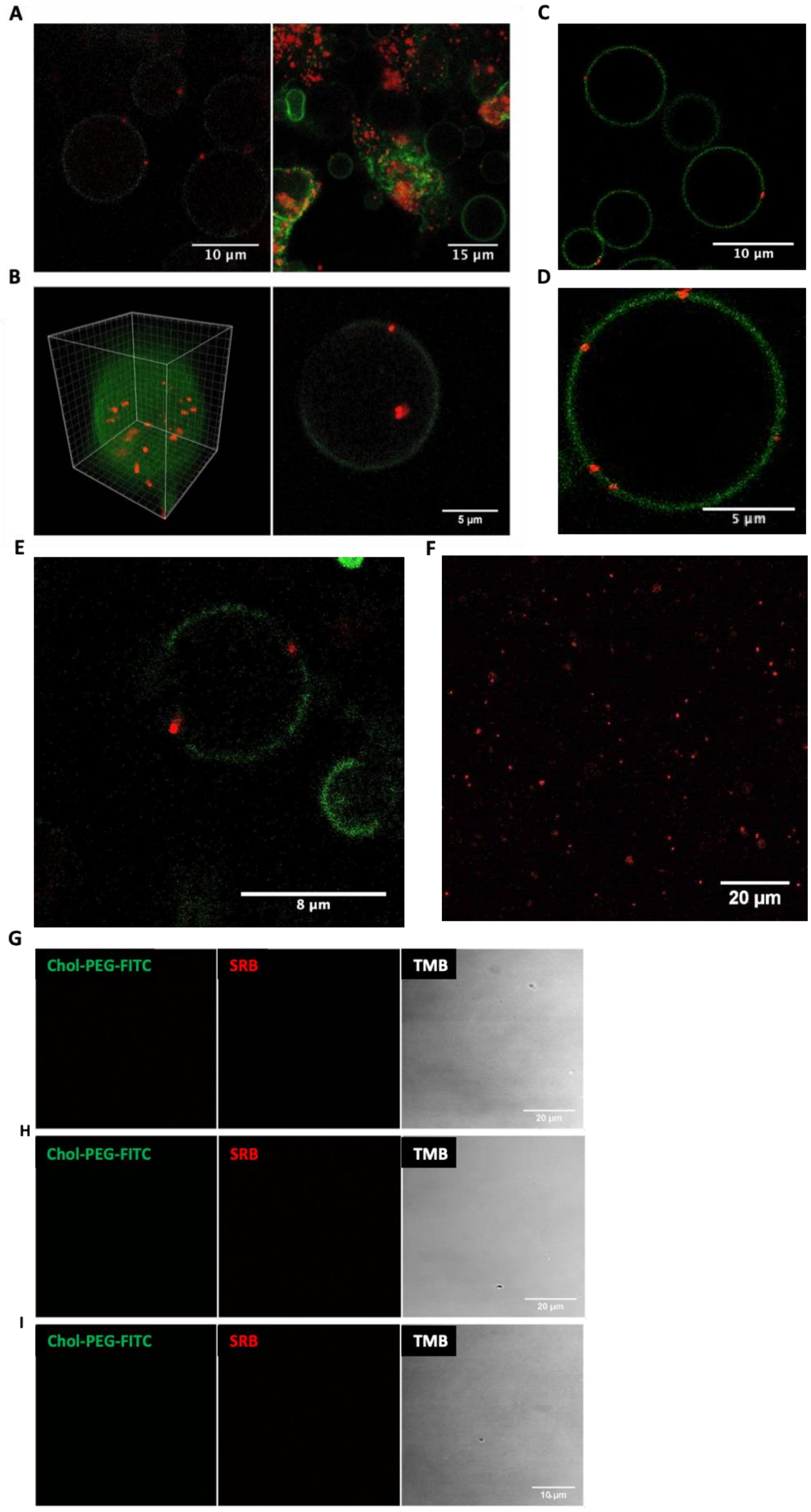

Figure S12. A) CLSM micrographs of SRB-loaded polymersomes in E-GPMVs after formation on HepG2 cells. Green: GFP fused Lck Tyrosine kinase (Lck-GFP). Red: SRB signal. B) 3D reconstructions of polymersome-loaded E-GPMVs (left) and single plane projection (right). Red: SRB signal. Green: Lck-GFP signal. C,D.) CLSM micrographs of isolated SRB polymersomes in E-GPMVs after isolation from HepG2 cells. Green: Lck-GFP Red: SRB signal. E) Stability of SRB-loaded polymersomes inside E-GPMVs after 7 days

storage at room temperature by using CLSM. Green: Lck-GFP membrane protein. Red: SRB-loaded polymersomes. F.) CLSM micrograph of SRB-loaded polymersomes in solution (0.01 mg mg mL-1, PBS, pH=7,4). G), H) and I) CLSM micrographs of empty polymersomes (1 mg mL-1) measured in buffer solution (PBS, pH=7,4). Green: Lck-GFP, Red: SRB.

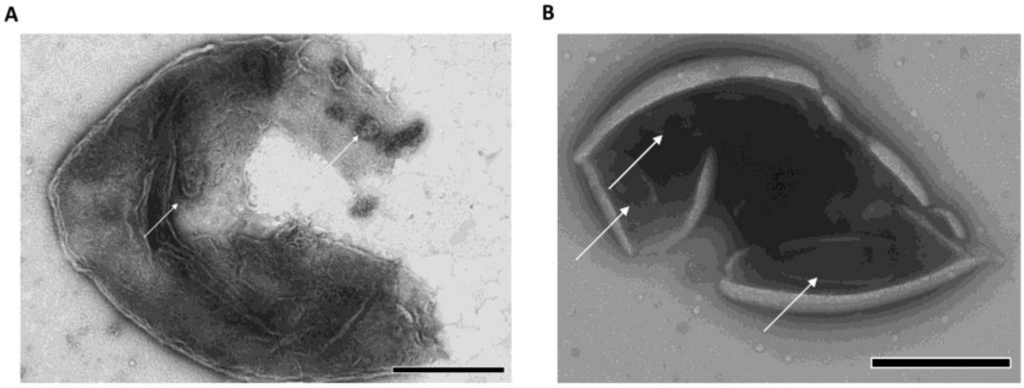

Figure S13. A) TEM micrograph of $PMOXA_6$-$PDMS_{44}$-$PMOXA_6$ polymersome E-GPMVs in HEPES pH 7.4. Scale bar 500 nm. Arrowheads: Polymersomes. B) TEM micrograph of $PMOXA_6$-$PDMS_{44}$-$PMOXA_6$ polymersome E-GPMVs. Scale bar 500 nm. Arrowheads: Polymersomes.

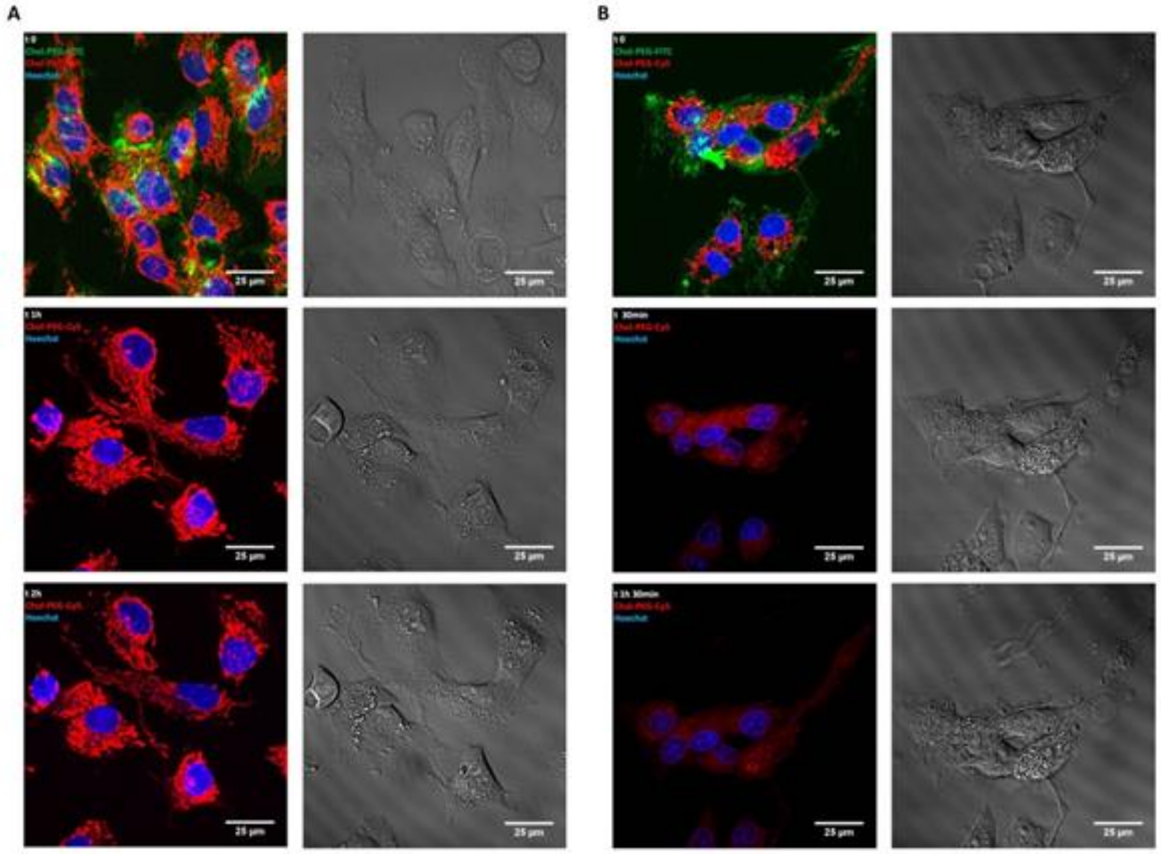

Figure S14. A) CLSM micrographs of intracellular membranes without inducing GPMV formation (HepG2 cells not exposed to vesiculation agents in DMEM, 5% FCS). Left: Maximum projection of HepG2 cells stained with Hoechst (Blue), Chol-PEG5000-Cy5 (Red) and Chol-PEG5000-FITC (Green). Right: Transmission channel. B) CLSM micrographs of

intracellular membrane reorganization during the vesiculation process – HepG cells exposed to the vesiculation buffer. Left: Maximum projection of HepG2 cells stained with Hoechst (Blue), Chol-PEG5000-Cy5 (Red) and Chol-PEG5000-FITC (Green). Right: Transmission channel.

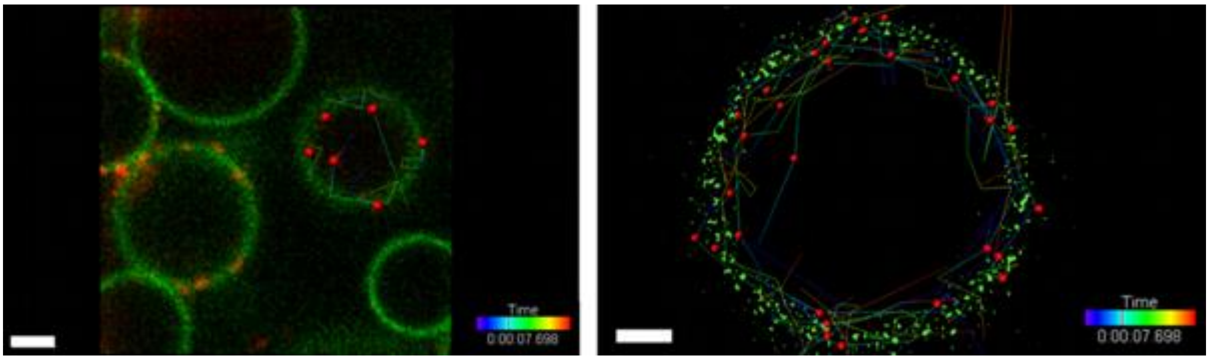

Figure S15. Particle tracking analysis of SRB-loaded polymersomes (Red) within E-GPMVs containing Chol-PEG5000-FITC in their membrane (Green). Left: Example of particle analysis by Imaris. Red signal superposed by Spheres. Right: Intra GPMVs diffusion SRB-loaded polymersomes. Single plane projections of particle tracking at different time points. Scale bar = 2 µm. Time span: 23 s.

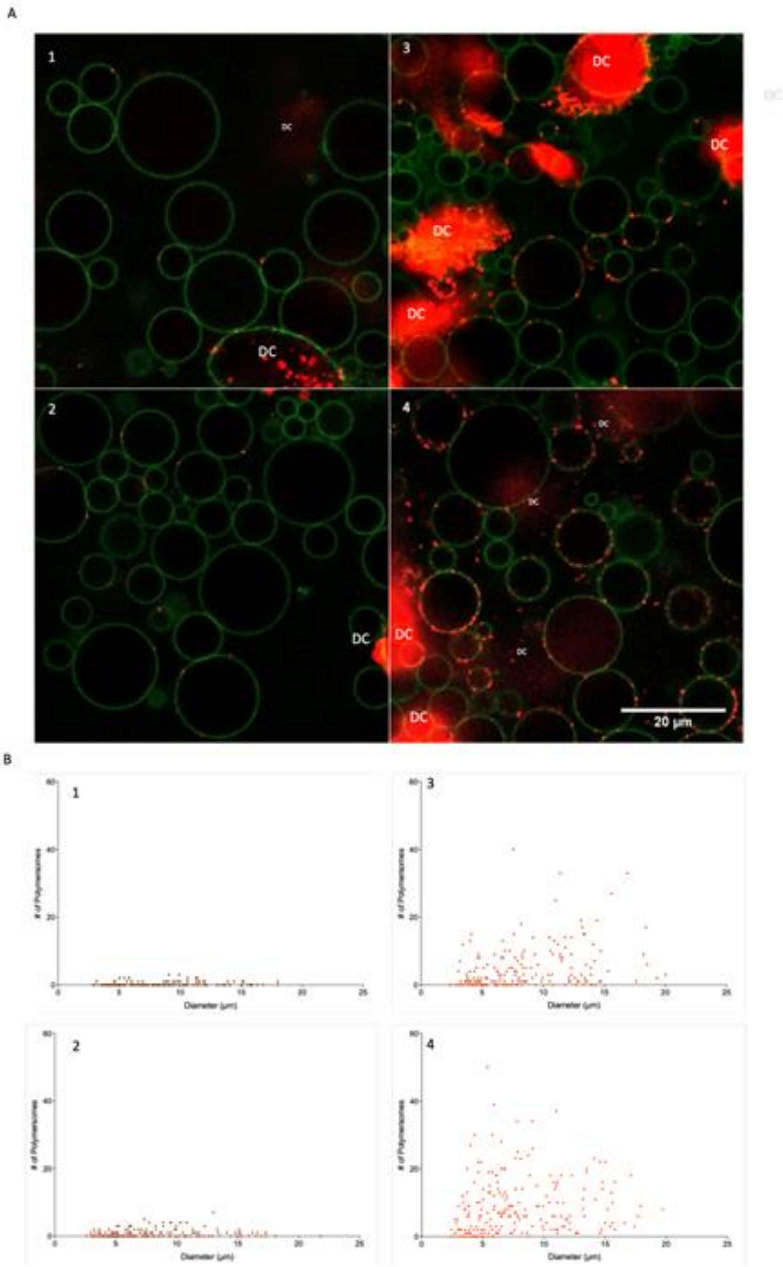

Figure S16. A) Single plane projections of polymersome-loaded E-GPMVs with Chol-PEG5000-FITC in their membrane for different initial concentrations of polymersomes: 1) 0.5 mg mL^{-1}, 2) 1.0 mg mL^{-1}, 3) 2 mg mL^{-1} and 4) 3 mg mL^{-1}. Red: Oversaturated areas represent donor cells loaded with SRB polymersomes (DC). Red: SRB-loaded polymersomes. Green: Chol-PEG5000-FITC. B) Evaluation of single plane projections of E-GPMVs loaded with different concentrations of polymersomes: 1) 0.5 mg mL^{-1}, 2) 1.0 mg mL^{-1}, 3) 2 mg mL^{-1} and 4) 3 mg mL^{-1}.

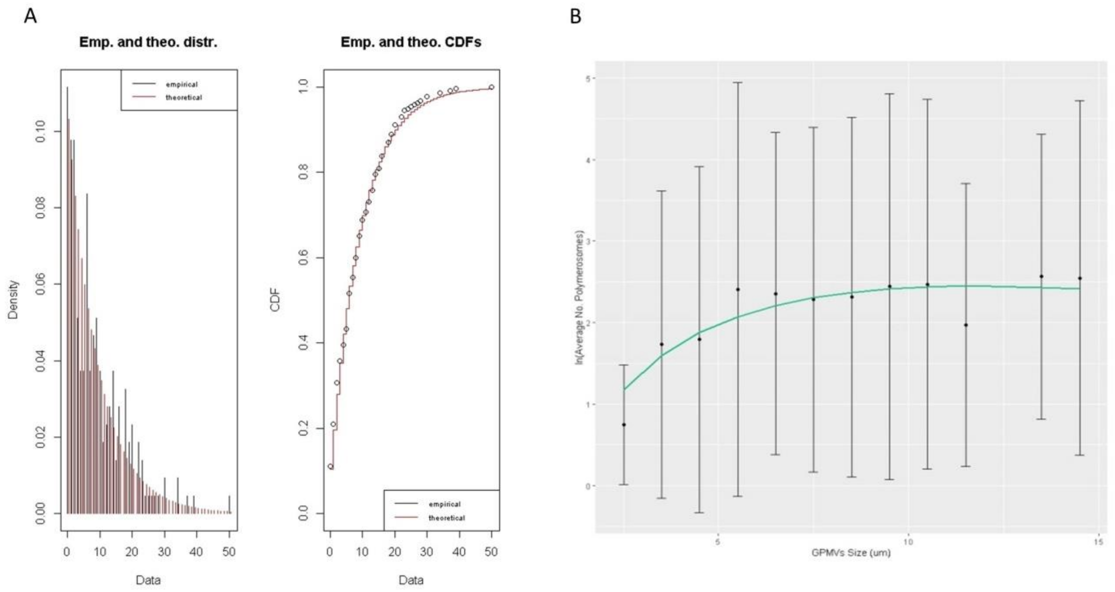

Figure S17. A) Empirical and theoretical distribution of the number of polymersomes/E-GPMVs (single plane CLSM data). Probability density function (left) and cumulative density function (right). B) Mean number of polymersomes/E-GPMVs in single plane projections of CLSM micrographs and fit with a log-log function of E-GPMVs sizes. Initial concentration of polymersomers used for cell up-take was 3 mg mL^{-1}.

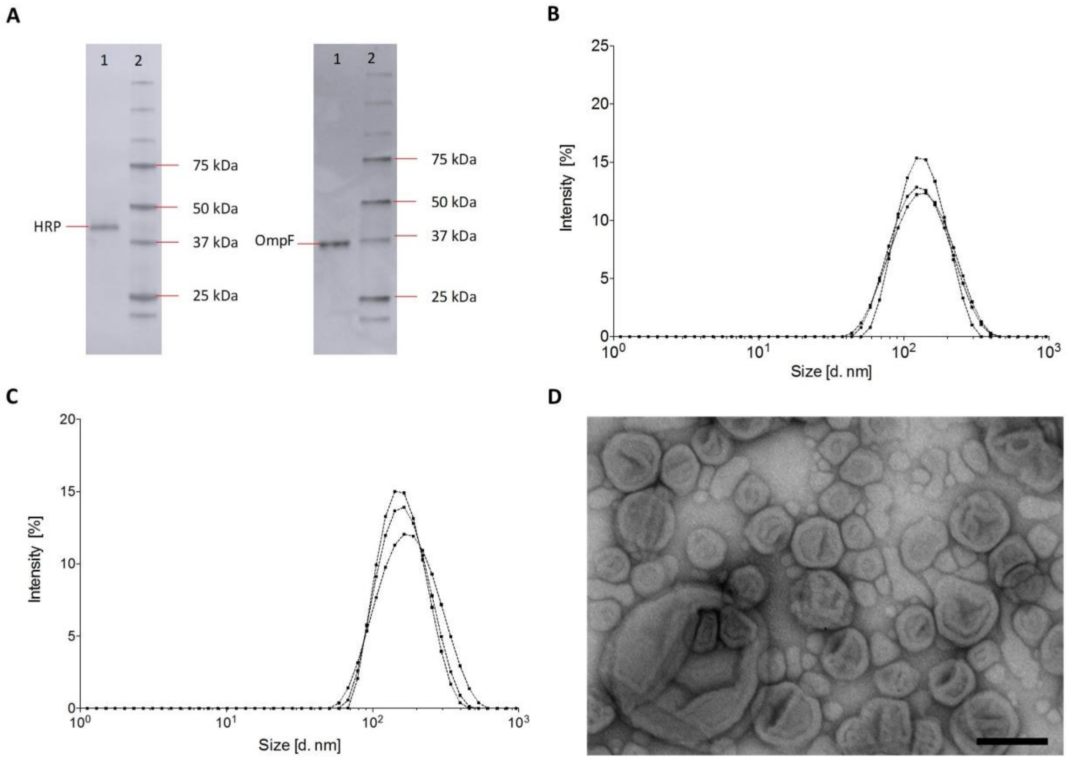

Figure S18. A) 4-15% Simply blue (Coomassie) stained SDS-PAGE gel of (left) HRP, Lane 1: HRP. Lane 2: Precision Plus BioRad Protein Ladder and (right) OmpF K89 R270. Lane 1:

OmpF K89 R270 Lane 2: Precision Plus BioRad Protein Ladder. B) Intensity distribution by DLS of catalytic compartments based on HRP-loaded $PMOXA_6$-$PDMS_{44}$-$PMOXA_6$ polymersomes equipped with OmpF, and measured in PBS pH 7.4 (n=3). C) Intensity distribution of DLS of catalytic compartments based on HRP-loaded $PMOXA_6$-$PDMS_{44}$-$PMOXA_6$ polymersomes without OmpF, and measured in PBS pH 7.4 (n=3). D) TEM micrograph of $PMOXA_6$-$PDMS_{44}$-$PMOXA_6$ polymersomes loaded with HRP and without OmpF. Scale bar represents 500 nm.

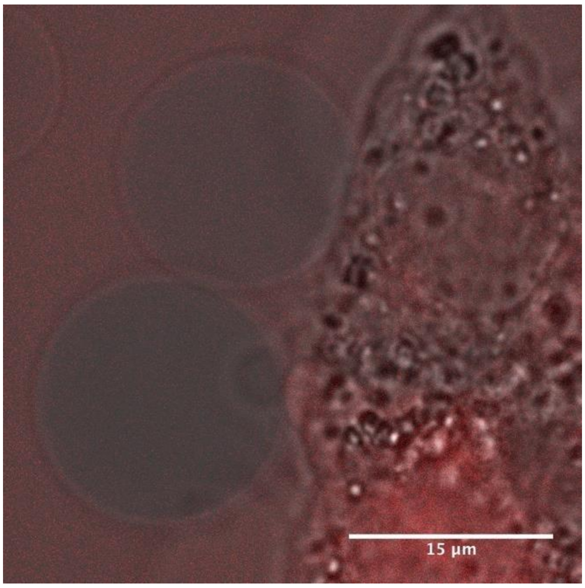

Figure S19. Amplex UltraRed reaction in E-GPMVs without AOs. Red: Resorufin-like product.

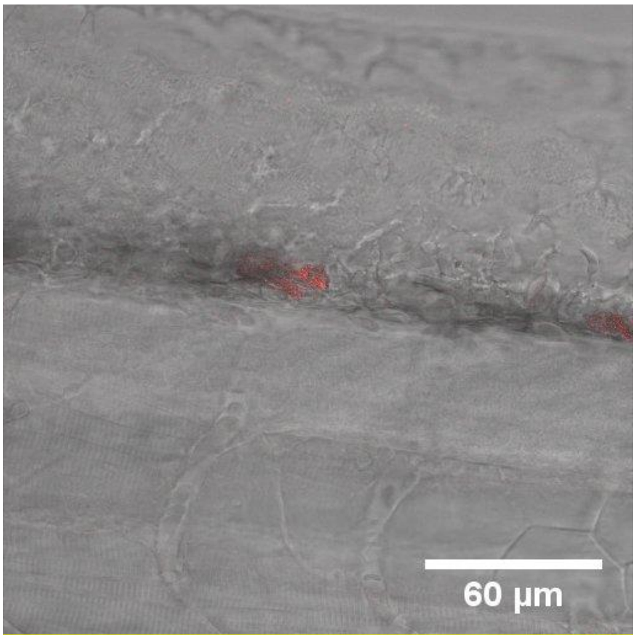

Figure S20. Melanocytes with autofluorescence. Individual melanocytes were selected as control for photo bleaching experiments.

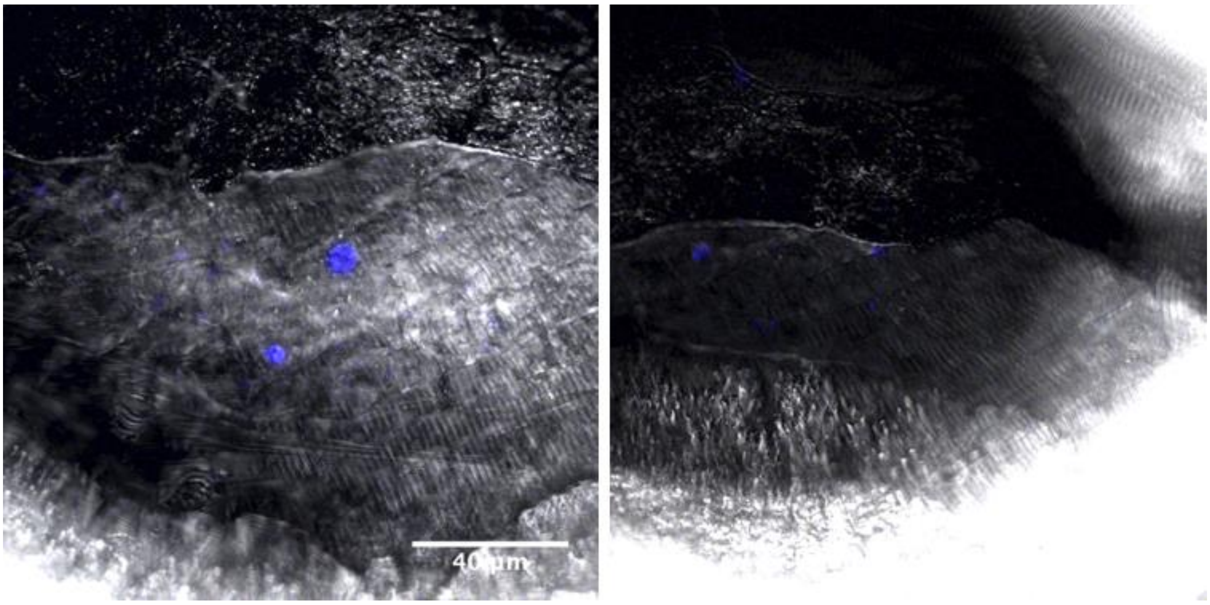

Figure S21. MAX Projections of CLSM *in vivo* analysis. ZFE peritoneal injections with GPMVs equipped with Chol-PEG5000 and CTDR. Blue: CTDR.

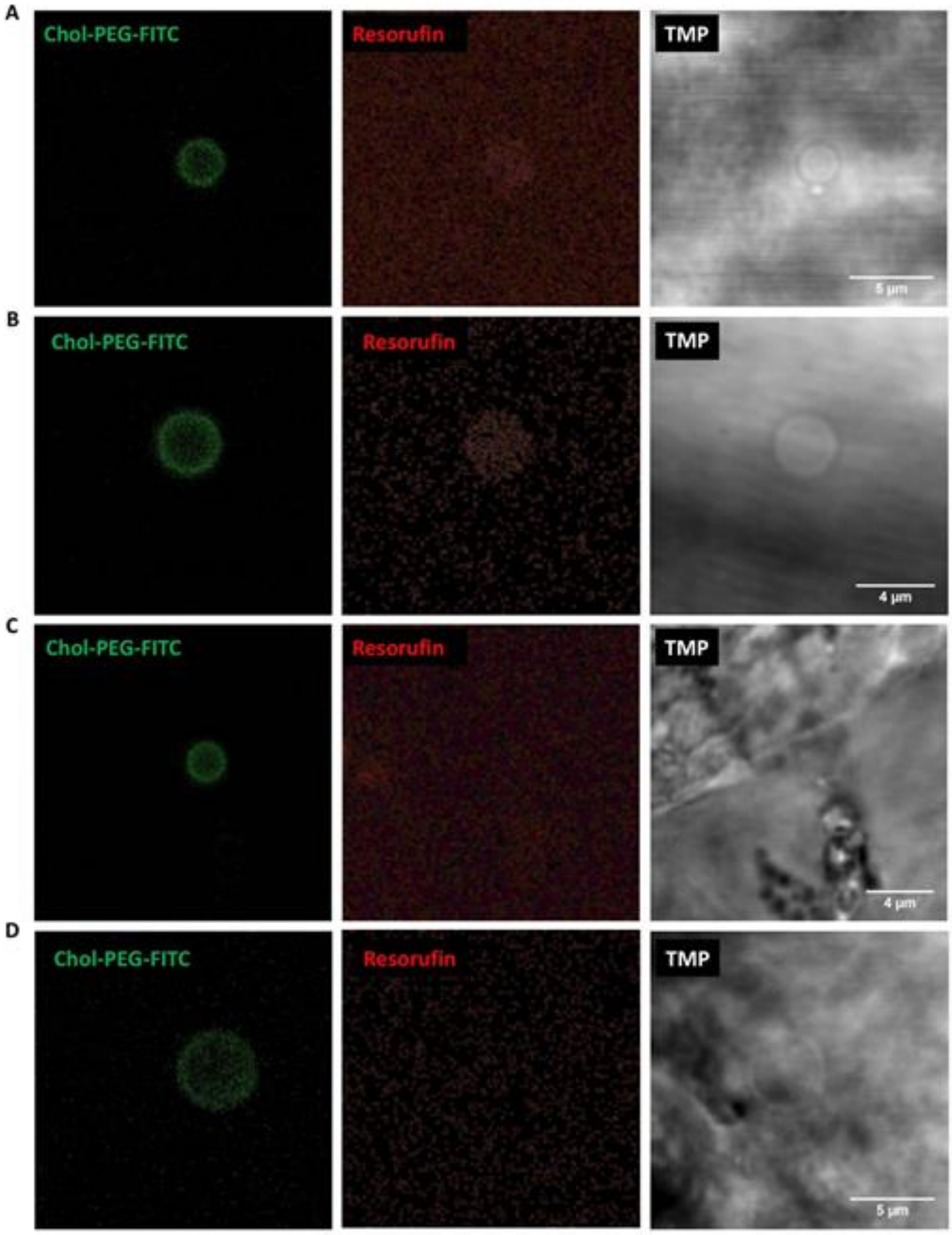

Figure S22. *In vivo* functionality of MFs in ZFE. Red: Resorufin-like product. Green: Chol-PEG5000-FITC. A) and B) MFs loaded with AOs based on HRP-loaded polymersomes with OmpF. C) and D) MFs loaded with AOs based on HRP-loaded polymersome without OmpF.

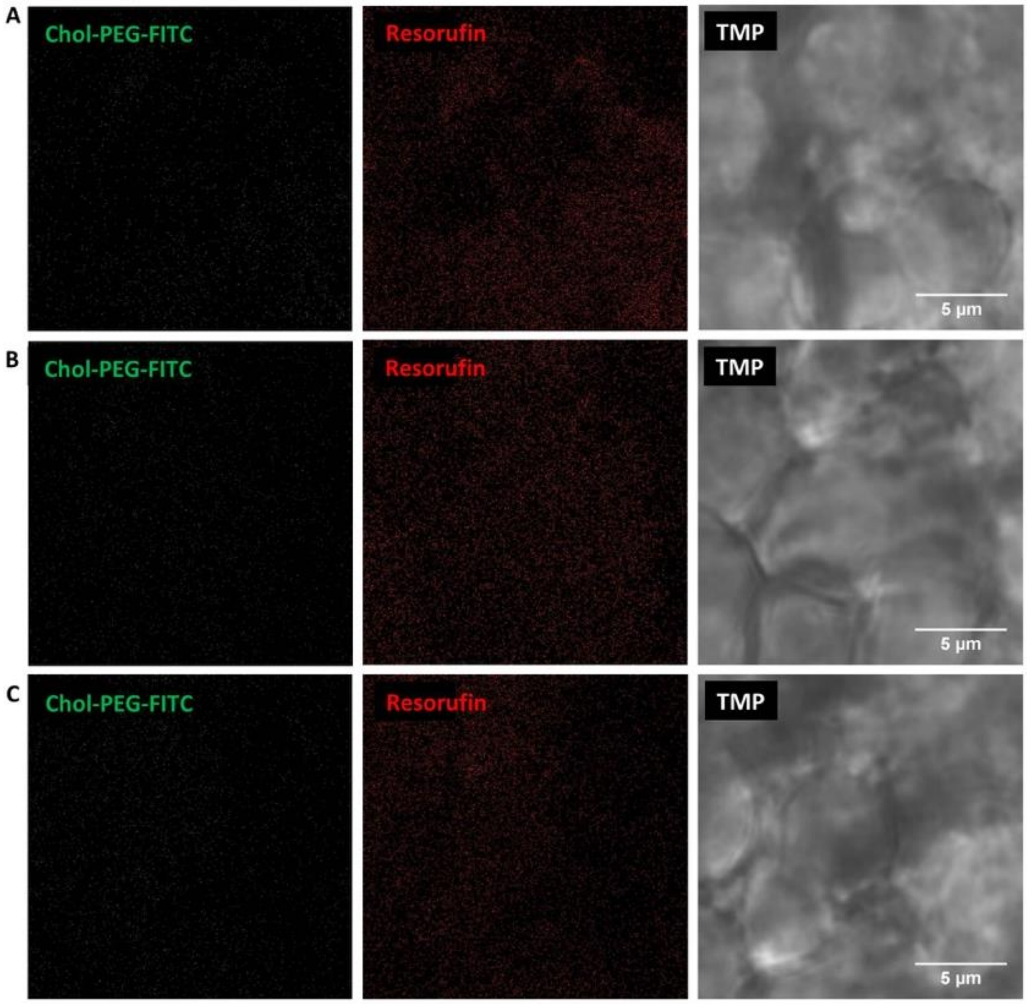

Figure S23. A), B) and C) ZFE injected with Amplex UltraRed and H_2O_2. Red: Resorufin-like product.

Supplementary Movies:

Movie S1. DSPC-loaded liposomes in E-GPMVs. Red: SRB signal. Green: GFP-Lck Tyrosine Kinase signal.

Movie S2. DSPS-loaded liposomes in E-GPMVs. Red: SRB signal. Green: GFP-Lck Tyrosine Kinase signal.

Movie S3. SRB-loaded PMOXA-PDMS-PMOXA polymersomes in E-GPMVs. Red: SRB signal. Green: GFP-Lck Tyrosine Kinase signal.

Movie S4. SRB-loaded PMOXA-PDMS-PMOXA polymersomes in E-GPMVs. Red: SRB signal. Green: GFP-Lck Tyrosine Kinase signal.

Movie S5. Diffusion of SRB-loaded PMOXA-PDMS-PMOXA polymersomes in E-GPMVs. Particle tracking analysis. Red: SRB signal. Green: GFP-Lck Tyrosine Kinase signal.

Movie S6. Stability of SRB-loaded PMOXA-PDMS-PMOXA polymersomes in GPMVs after storage at RT for 7 Days. Red: SRB signal. Green: GFP-Lck Tyrosine Kinase signal.

Movie S7. SRB-loaded PMOXA-PDMS-PMOXA polymersomes in E-GPMVs at different concentrations. Red: SRB signal. Green: Chol-PEG5000-FITC signal.

Movie S8. SRB-loaded PMOXA-PDMS-PMOXA polymersomes in E-GPMVs containing water soluble E-GFP. Red: SRB signal. Green: E-GFP signal.

Movie S9. E-GPMVs compartmentalizing OmpF equipped AOs converting Amplex UltraRed into resorufin-like product. Red: Resorufin-like product.

Movie S10. MFs compartmentalizing AOs deficient of OmpF. Red: Resorufin-like product.

Movie S11. Chol-PEG-5000-FITC equipped E-GPMVs loaded with CTDR in ZFE circulation. Green: Chol-PEG-FITC

Movie S12. Chol-PEG-5000-CLS equipped E-GPMVs loaded with water soluble RFP in ZFE circulation. Red: RFP

Movie S13. Chol-PEG5000-FITC equipped E-GPMVs loaded with CTDR in ZFE circulation. Blue: CTDR Green: Chol-PEG-FITC

Movie S14. Chol-PEG-5000-CLS equipped E-GPMVs loaded with water soluble RFP in ZFE circulation. Red: RFP. Green: GFP ZFE vasculature.

References:

[1] X. Chen, X. Zhang, H.-Y. Wang, Z. Chen, F.-G. Wu, *Langmuir* **2016**, *32*, 10126.
[2] F. M. Boyce, N. L. Bucher, *Proc. Natl. Acad. Sci. U.S.A.* **1996**, *93*, 2348.
[3] T. Einfalt, D. Witzigmann, C. Edlinger, S. Sieber, R. Goers, A. Najer, M. Spulber, O. Onaca-Fischer, J. Huwyler, C. G. Palivan, *Nature Commun.* **2018**, *9*, 1127.
[4] F. Itel, M. Chami, A. Najer, S. Lörcher, D. Wu, I. A. Dinu, W. Meier, *Macromolecules* **2014**, *47*, 7588.
[5] Bolte S., Cordelières F. P., *Journal of Microscopy* **2006**, *224*, 213.
[6] C. Edlinger, T. Einfalt, M. Spulber, A. Car, W. Meier, C. G. Palivan, *Nano Lett.* **2017**, *17*, 5790.
[7] C. A. Di Buduo, L. S. Wray, L. Tozzi, A. Malara, Y. Chen, C. E. Ghezzi, D. Smoot, C. Sfara, A. Antonelli, E. Spedden, G. Bruni, C. Staii, L. De Marco, M. Magnani, D. L. Kaplan, A. Balduini, *Blood* **2015**, *125*, 2254.
